# Twelve new species and fifty-three new provincial distribution records of Aleocharinae rove beetles of Saskatchewan, Canada (Coleoptera, Staphylinidae)

**DOI:** 10.3897/zookeys.610.9361

**Published:** 2016-08-11

**Authors:** Jan Klimaszewski, David J. Larson, Myriam Labrecque, Caroline Bourdon

**Affiliations:** 1 Natural Resources Canada, Canadian Forest Service, Laurentian Forestry Centre, 1055 du P.E.P.S., P.O. Box 10380, Stn. Sainte-Foy, Québec, Quebec, Canada G1V 4C7; 2 P.O. Box 56, Maple Creek, Saskatchewan, Canada S0N 1N0

**Keywords:** Coleoptera, rove beetles, Staphylinidae, new distribution records, new species, Canada, Saskatchewan

## Abstract

One hundred twenty species of aleocharine beetles (Staphylinidae) are recognized in the province of Saskatchewan. Sixty-five new provincial records, including twelve new species and one new North American record, are presented. *Oligota inflata* (Mannerheim), a Palearctic species, is newly recorded for North America. The following twelve species are described as new to science: *Acrotona pseudopygmaea* Klimaszewski & Larson, **sp. n.**, *Agaricomorpha pulchra* Klimaszewski & Larson, **sp. n.** (new genus record for Canadian fauna), *Aleochara elisabethae* Klimaszewski & Larson, **sp. n.**, Atheta (Dimetrota) larsonae Klimaszewski & Larson, **sp. n.**, Atheta (Microdota) pseudopittionii Klimaszewski & Larson, **sp. n.**, Atheta (Microdota) spermathecorum Klimaszewski & Larson, **sp. n.**, *Atheta* (*sensu lato*) *richardsoni* Klimaszewski & Larson, **sp. n.**, *Brachyusa saskatchewanae* Klimaszewski & Larson, **sp. n.**, *Dochmonota langori* Klimaszewski & Larson, **sp. n.**, *Dochmonota simulans* Klimaszewski & Larson, **sp. n.**, *Dochmonota websteri* Klimaszewski & Larson, **sp. n.**, and *Oxypoda domestica* Klimaszewski & Larson, **sp. n.** Colour images of habitus and black and white images of the median lobe of the aedeagus, spermatheca, and tergite and sternite VIII are presented for all new species, *Oligota inflata* Mannerheim and *Dochmonota rudiventris* (Eppelsheim). A new synonymy is established: *Tetralina filitarsus* Casey, **syn. n.** = *Tetralina helenae* Casey, now placed in the genus *Brachyusa* Mulsant & Rey.

## Introduction

Aleocharinae beetles are the most species-rich subfamily of rove beetles (Staphylinidae) with 515 species recorded in the most recent catalog of the Coleoptera of Canada (Bousquet et al. 2013). This number is constantly increasing as new treatments of this group are published. Webster et al. (2016b) added 27 new species to the Canadian fauna from the province of New Brunswick, and Klimaszewski et al. (2015a) added two new Canadian records of species previously known from the USA, so the total number now stands at 544 species excluding the new species treated here. Aleocharinae is still one of the poorest known subfamily of rove beetles in Canada, although enormous progress has been made in investigating this group in the last two decades, especially in eastern Canada (Klimaszewski et al. 2011, Webster et al. 2016a, b). Western and northern Canada (Manitoba to British Columbia, and the three territories) (Klimaszewski et al. 2015a), however, remain poorly studied except for a few localities in coastal British Columbia (Klimaszewski and Winchester 2002, McLean et al. 2009a, b) and in the Yukon (Klimaszewski et al. 2008b, 2012). Thus the full distribution of many Canadian species is not known because of large gaps in sampling intensity (Klimaszewski et al. 2015a). Improved sampling of Staphylinidae, especially Aleocharinae, is needed to establish baseline biodiversity composition in areas of the country where ecosystems are undergoing rapid change due to resource extraction and climate change (Klimaszewski et al. 2015a). This paper contributes to improving baseline biodiversity knowledge of aleocharine beetles in the province of Saskatchewan (SK) by providing 65 new provincial species records including one new North American record and 12 species new to science.

## Materials and methods

Almost all specimens in this study were dissected to examine the genital structures. Extracted genital structures were dehydrated in absolute alcohol, mounted in Canada balsam on celluloid micro-slides, and pinned with the specimen from which they originated. Images of the entire body and the genital structures were taken using an image processing system (Nikon SMZ 1500 stereoscopic microscope; Nikon Digital Camera DXM 1200F, and Adobe Photoshop software).

Morphological terminology mainly follows that used by Seevers (1978) and Klimaszewski et al. (2011). The ventral side of the median lobe of the aedeagus is considered to be the side of the bulbus containing the foramen mediale, the entrance of the ductus ejaculatorius, and the adjacent ventral side of the tubus of the median lobe with the internal sac and its structures (this part is referred to as the parameral side in some recent publications); the opposite side is referred to as the dorsal part. In the species descriptions, microsculpture refers to the surface of the upper forebody (head, pronotum and elytra).

Tribes, genera and species within genera are arranged alphabetically in the text and in the Table 1.

**Table 1.** Species of Aleocharinae recorded from Saskatchewan and their provincial and territorial distribution within Canada. Provinces and territories in bold denote new records given in the present publication. Species marked with (†) indicate adventive species and species marked with (*) are Holarctic.

**ALEOCHARINI**	
*Aleochara assiniboin* Klimaszewski	BC, MB, ON, SK, YT
*Aleochara bilineata* Gyllenhal†	AB, BC, MB, NB, NF, NS, ON, PE, QC, SK; USA: New England states
*Aleochara bimaculata* Gravenhorst	AB, BC, LB, MB, NB, NF, NS, ON, QC, SK, NT; USA: widespread
***Aleochara elisabethae* Klimaszewski & Larson, sp. n.**	**SK**
*Aleochara gracilicornis* Bernhauer	BC, MB, NB, NS, NT, ON, QC, SK; USA: widespread
***Aleochara inexpectata* Klimaszewski**	NB, NS, ON, QC, **SK**; USA: MI, WI
*Aleochara lacertina* Sharp	AB, BC, MB, NB, NF, NS, ON, QC, SK; USA: widespread
*Aleochara laramiensis* (Casey)	BC, SK; USA: CO, WY
*Aleochara lata* Gravenhorst†	BC, MB, ON, QC, SK, YT; USA: widespread
***Aleochara rubricalis* (Casey)**	BC, ON, **SK**; USA: CA, AZ
*Aleochara sekanai* Klimaszewski	AB, LB, MB, NB, NT, ON, SK, YT; USA: AK
***Aleochara speculicollis* Bernhauer**	AB, ON, QC, **SK**: USA: CA, CO, AZ, MI, NV, TX
***Aleochara suffusa* (Casey)**	AB, BC, MB, QC, **SK**; USA: AK, AZ, CO, NM, WY
*Aleochara tahoensis* Casey	AB, BC, MB, NB, NS, NT, ON, SK, YT; USA: CA, CO, MT, NH, NM, NV, OR, WA
*Aleochara verna* Say	AB, BC, LB, MB, NB, NF, NS, ON, PE, QC, SK, YT; USA: widespread including AK
***Aleochara villosa* Mannerheim**†	AB, BC, NB, QC, **SK**; USA: AK, CA, OR, WA
*Tinotus morion* (Gravenhorst) † [now regarded as *Aleochara*]	AB, BC, NB, NF, NS, ON, QC, SK; USA: CT, NV
**ATHETINI**	
***Acrotona pseudopygmaea* Klimaszewski & Larson, sp. n.**	**SK**
*Acrotona recondita* (Erichson)	SK; USA: AR, CA, NH, NV, NY, PA
***Acrotona subpygmaea* (Bernhauer)**	NB, NS, ON, **SK**
***Amischa analis* (Gravenhorst)** †	LB, NB, NF, NS, ON, QC, PE, **SK**
*Atheta celata* (Erichson) *	BC, NB, NF, NS, QC, SK; USA: AK
***Atheta crenuliventris* Bernhauer**	LB, NB, NF, ON, QC, **SK**
*Atheta dadopora* C.G. Thomson *	AB, BC, LB, NB, NF, NS, ON, PE, SK, YT; USA: AK, NY, PA, RI
***Atheta districta* Casey**	AB, BC, LB, NB, NF, NS, ON, QC, **SK**
*Atheta fanatica* Casey	AB, BC, LB, NB, NS, QC, SK, YT; USA: AK, NV
***Atheta frosti* Bernhauer**	BC, LB, NB, NS, ON, QC, **SK**
*Atheta graminicola* (Gravenhorst) *	AB, BC, LB, MB, NB, NF, NT, ON, QC, SK, YT; USA: AK, OR
*Atheta klagesi* Bernhauer	AB, BC, NB, NF, NS, ON, PE, QC, SK, YT; USA: IA, ME, MN, NJ, NY, PA
***Atheta larsonae* Klimaszewski & Larson, sp. n.**	**SK**
*Atheta longicornis* (Gravenhorst) †	BC, NB, NF, NS, QC, SK; USA: CA, MN
*Atheta nigra* (Kraatz) †	SK
*Atheta platonoffi* Brundin*	AB, BC, LB, NB, NF, NS, ON, SK, YT; USA: AK
*Atheta prudhoensis* (Lohse)	BC, LB, NB, NF, NS, ON, QC, SK, YT; USA: AK, VT
***Atheta pseudometlakatlana* Klimaszewski & Godin**	YT, **SK**
***Atheta pseudopittionii* Klimaszewski & Larson, sp. n.**	SK
***Atheta pseudoschistoglossa* Klimaszewski & Webster**	BC, NB, **SK**; USA: AK
*Atheta recondita* (Erichson)	SK; USA: AR, CA, NH, NV, PA
***Atheta remulsa* Casey**	AB, BC, LB, NB, NF, NS, ON, QC, **SK**, YT
***Atheta riparia* Klimaszewski & Godin**	**SK**, YT
***Atheta richardsoni* Klimaszewski & Larson, sp. n.**	**SK**
***Atheta spermathecorum* Klimaszewski & Larson, sp. n.**	**SK**
***Atheta strigosula* Casey**	BC, LB, NB, NF, ON, QC, **SK**, YT; USA: NY
***Atheta subsinuata* (Erichson)** †	YT, **SK**
***Atheta terranovae* Klimaszewski & Langor**	LB, NB, NF, ON, **SK**, YT
*Atheta ventricosa* Bernhauer	AB, BC, LB, NB, NF, NS, ON, QC, SK, YT; USA: AK, DC, NC, NJ, NY, PA, VT
***Dinaraea angustula* (Gyllenhal)** †	AB, LB, NB, NF, NS, ON, PE, QC, **SK**, YT; USA: CA, NY
***Dinaraea pacei* Klimaszewski & Langor**	AB, BC, LB, NB, QC, **SK**, YT; USA: AK
***Dochmonota langori* Klimaszewski & Larson, sp. n.**	**SK**
***Dochmonota simulans* Klimaszewski & Larson, sp. n.**	**SK**
***Dochmonota websteri* Klimaszewski & Larson, sp. n.**	**SK**
***Earota dentata* (Bernhauer)**	AB, BC, MB, NB, NF, NS, ON, QC, **SK**, YT; USA: AK
*Lypoglossa franclemonti* Hoebeke	AB, MB, NB, NF, NS, NT, ON, QC, SK, YT; USA: NY, VT
***Mocyta breviuscula* (Mäklin)**	AB, BC, LB, NB, NF, NS, ON, QC, **SK**, YT; USA: AK
***Mocyta discreta*** (Casey)	ON, QC, SK; USA: IA, MN
***Mocyta spahgnorum* Klimaszewski & Webster**	NB, NF, ON, QC, **SK**
***Nehemitropia lividipennis* (Mannerheim)** †	NB, NF, NS, ON, PE, QC, **SK**; USA: CA, LA, MA, MN, NE, NM, NY, PA, VT, TX
*Philhygra botanicarum* (Muona) *	BC, LB, NB, NF, NS, ON, SK, YT
***Philhygra falcifera* Lohse**	MB, **SK**
*Philhygra jarmilae* Klimaszewski & Langor	NB, NF, ON, SK, YT
*Philhygra ripicoloides* Lohse	NF, NT, SK, YT
*Philhygra rostrifera* Lohse	LB, NT, SK, YT; USA: AK
*Philhygra sinuipennis* Klimaszewski & Langor	NB, LB, NF, SK, YT
***Philhygra subpolaris* (Fenyes)**	AB, **SK**; USA: AZ
*Philhygra terrestris* Klimaszewski & Godin	NB, SK, YT
***Schistoglossa blatchleyi* (Bernhauer & Scheerpeltz)**	MB, NB, NT, ON, QC, **SK**, YT; USA: AK, IN
*Seeversiella globicollis* (Bernhauer)	AB, BC, NB, NF, NS, ON, QC, SK; USA: AZ, CO, ID, MN, MT, NH, SD, WI
***Strigota ambigua* (Erichson)**	LB, NB, NS, NF, ON, PE, **SK**, YT; USA: CA, CO, CT, IA, KS, MO, NC, NJ, NM, NY, TX
***Strigota obscurata* Klimaszewski & Brunke**	NB, ON, **SK**
**AUTALIINI**	
***Autalia rivularis* (Gravenhorst)** †	AB, BC, LB, NB, NF, NS, ON, QC, **SK**; USA: CA, MI, MN, NH, NY, OR
**FALAGRINI**	
***Falagria caesa* Erichson**†	AB, NB, ON, QC, **SK**; USA: MA to VA, UT
*Falagria dissecta* Erichson	AB, BC, MB, NB, NS, ON, QC, SK; USA: widespread
***Myrmecocephalus arizonicus* (Casey)**	AB, BC, **SK**
**GYMNUSINI**	
*Gymnusa campbelli* Klimaszewski	MB, NB, NF, NT, ON, QC, SK, YT; USA: AK
**HOMALOTINI**	
***Agaricochara pulchra* Klimaszewski & Larson, sp. n.**	**SK**
*Gyrophaena affinis* Mannerheim	BC, MB, NB, NF, NS, ON, QC, SK; USA: widespread
*Gyrophaena criddlei* Casey	LB, MB, NB, ON, SK, YT
*Gyrophaena insolens* Casey	BC, LB, MB, NB, NF, ON, SK; USA: MI
*Gyrophaena keeni* Casey	AB, BC, LB, NB, NF, ON, QC, SK, YT; USA: FL, MA, MT, NH, NY, TN, WA
***Gyrophaena lobata* Casey**	NB, **SK**; USA: DC, IL, IN, KS, MI, WI
*Gyrophaena uteana* Casey	AB, BC, NB, ON, QC, SK; USA: CA, CO, UT
***Gyrophaena subnitens* Casey (NCR)**	MB, **SK**; USA: IL, KS, ME, MN, MO, WI
***Leptusa gatineauensis* Klimaszewski & Pelletier**	AB, BC, NB, NF, NS, ON, QC, **SK**
**HYPOCYPHTINI**	
***Cypha crotchi* (Horn)**	AB, BC, **SK**
***Cypha inexpectata* Klimaszewski & Godin**	ON, YT, **SK**
***Oligota inflata* (Mannerheim)† (NPR, NCR, NAR)**	**SK**
**LOMECHUSINI**	
*Xenodusa reflexa* (Walker)	AB, BC, MB, NB, NS, QC, ON, SK
***Zyras obliquus*** (Casey)	AB, BC, MB, NB, NF, NS, ON, QC, **SK**; USA: MI, MO, NH, NY, OR
**MYLLAENINI**	
*Myllaena arcana* Casey	AB, LB, NB, NF, NS, ON, QC, SK; USA: AL, FL, IA, IL, MA, NH, NJ
*Mylaena insomnis* Casey	AB, BC, LB, MB, NB, NF, NS, NT, ON, QC, SK, YT; USA: AK, ID, MA, MN, WI
**OXYPODINI**	
*Cratarea suturalis* (Mannerheim) †	BC, LB, NB, NS, ON, SK; USA: IL, MA, MO, PA, SC, VA, VT
*Devia prospera* (Erichson) *	AB, BC, LB, MB, NB, NT, ON, SK, YT; USA: AK, CO, MI, MN, NM, OR, SD, UT, WA, WY
***Gnathusa eva* Fenyes**	AB, BC, **SK**, YT; USA: CA
***Hylota ochracea* Casey**	NB, NS, NT, ON, QC, **SK**; USA: NY
*Ocyusa canadensis* Lohse	NB, NF, ON, SK, YT; USA: AK
*Oxypoda canadensis* Klimaszewski	AB, MB, LB, NF, NT, ON, QC, SK, YT; USA: AK
***Oxypoda demissa* Casey**	LB, NB, NF, NS, ON, QC, **SK**, YT
***Oxypoda domestica* Klimaszewski & Larson, sp. n.**	**SK**
*Oxypoda grandipennis* (Casey)	AB, BC, LB, NB, NF, NS, ON, QC, SK, YT; USA: AK, NH
***Oxypoda irrasa* Mäklin**	AB, **SK**, YT; USA: AK, OR
*Oxypoda lacustris* Casey	AB, BC, LB, MB, NB, NF, NS, NT, ON, QC, SK, YT; USA: AK
***Oxypoda manitobae* Casey**	BC, MB, **SK**; USA: CO
*Oxypoda orbicollis* Casey	AB, LB, NB, NS, ON, QC, SK, YT; USA: WI
*Oxypoda pseudolacustris* Klimaszewski	AB, NB, NF, NS, ON, QC, SK
***Parocyusa fuliginosa* (Casey)**	LB, ON, **SK**; USA: MA, NC, PA
*Tachyusa obsoleta* Casey	BC, NB, SK
**PLACUSINI**	
***Placusa incompleta* Sjöberg** †	AB, BC, NB, NF, NS, ON, QC, **SK**; USA: WA
***Placusa pseudosuecica* Klimaszewski**	AB, BC, ON, QC, **SK**
***Placusa tachyporoides* (Waltl)** †	AB, BC, NB, NS, ON, QC, **SK**; USA: CA, MA
***Placusa tacomae* Casey**	AB, BC, NB, NF, NS, NT, ON, QC, **SK**, YT; USA: AZ, MA, WA, WI
***Placusa vaga* Casey**	BC, NB, NS, NT, ON, QC, **SK**, YT; USA: CA
**SILUSINI**	
***Silusa californica* Bernhauer**	AB, BC, NB, NF, NS, NT, QC, ON, **SK**, YT; USA: AK, CA, MN
**TACHYUSINI**	
***Brachyusa helenae* (Casey)**	LB, NB, NF, NT, ON, **SK**, YT; USA: AK, MT
***Brachyusa saskatchewanae* Klimaszewski & Larson, sp. n.**	**SK**
*Gnypeta caerula* (C.R. Sahlberg) *	AB, BC, LB, MB, NB, NF, NS, NT, ON, PE, QC, SK, YT; USA: AK
*Gnypeta carbonaria* (Mannerheim)	AB, MB, NB, NF, NT, ON, QC, SK; USA: AK
*Gnypeta dentata* Klimaszewski	AB, NT, SK
***Gnypeta minuta* Klimaszewski & Webster**	NB, **SK**
***Gnypeta saccharina* Klimaszewski & Webster**	NB, **SK**
*Gnypeta sellmani* Brundin	LB, MB, NF, NT, QC, SK, YT; USA: AK

### Major habitat characterization

Almost all collections reported here were made in southwestern Saskatchewan and adjacent Alberta. This area is in the Mixed Grassland and Cypress Upland Ecoregions of the Prairies Ecozone (Ecological Stratification Working Group 1995). The Mixed Grasslands are a semiarid northern portion of the shortgrass prairie of the North American Great Plains. Summer moisture deficits promote the dominance of grasses (especially spear, blue gramma and wheat grasses) and a variety of low herbs and shrubs including sagebrush and cactus. This grassland encircles an upland area known as the Cypress Uplands. These uplands rise rather abruptly from the plains in the west to their highest elevations of almost 1500 m in SE Alberta and adjacent SK, then gradually become lower towards the east. Much of the uplands are treed with lodgepole pine, white spruce and aspen with open areas dominated by rough fescue grass and shrubby cinquefoil. The 1000 m contour was arbitrarily chosen as the boundary between these two zones for the actual boundary is complex with interdigitation of habitats such as trees and mesic plants following stream courses and valleys out into the grasslands and conversely dry grassland species occur on ridges and south-facing slopes well above the 1000 m contour. The most frequently referenced collection site is the Larson Ranch. This is located on the boundary of these two ecozones with the 1000 m contour running through the farmyard. Collections here are from a variety of habitats including: aspen or maple woodlands; fescue-cinquefoil or mixed grasslands; stream and pond margins; and on various soil types including arid tills and bedrock clays. Many ranch collections are from habitats of domestic or agricultural origin such as compost and manure piles, livestock housing or associated with exotic plants.

Other habitats within the area from which aleocharines have been collected include sand hills and saline ponds of closed drainage basins, both of which occur mainly to the north of the Cypress Hills, stream margins, and springs and fens that are common in the Cypress Hills. Considerable collecting has been done around large reservoirs. Accumulated plant material along the reservoir water lines (wrack) is often rich in beetles but windward shores (the lee shore of mariners) are often rich collecting sites as flying insects that fall into water are blown onto these shores and can sometimes be found in large numbers pulling themselves up onto the beach. Such insects are referred to as occurring in drift. Species found in wrack may in fact be in their normal habitat, but those recorded from drift are probably vagrants, but they do indicate presence and time of year of dispersal.

The low annual precipitation in the region means that a state of drought or near drought occurs frequently. Aleocharines occur mainly in moist environments, thus the majority of Mixed Grassland collections are from sites with moisture such as margins of water bodies or from moist habitats such as carrion and manure (which is very abundant due to the high populations of cattle). Carrion and manure are rich staphylinid habitat but they promote the widespread synanthropic species and a few of our new records come from these habitats. Mushrooms and other fungi, especially as they age and decay, are rich habitats but again irregularity in precipitation means that occurrence and duration of such habitats is very unpredictable over the season and from year to year. Higher levels of precipitation and lower evapotranspiration in the Cypress Uplands produce a wider and more consistent array of moist habitats and this is where we found the richest aleocharine fauna.

### Depository/institutional abbreviations

BGC
 Benoit Godin collection, Whitehorse, Yukon Territory, Canada 


CNC
 Canadian National Collection of Insects, Arachnids and Nematodes, Agriculture and Agri-Food Canada, Ottawa, Ontario, Canada 


LFC
 Natural Resources Canada, Canadian Forest Service, Laurentian Forestry Centre, R. Martineau Insectarium, Quebec City, Quebec, Canada 


DLC
 David Larson collection, Maple Creek, Saskatchewan, Canada 


USNM
 United State National Museum, Washington, D.C., USA 


### Abbreviations of Canadian Provinces and Territories

AB – Alberta


BC – British Columbia


LB – Labrador


MB – Manitoba


NB – New Brunswick


NF – Newfoundland


NS – Nova Scotia


NT – Northwest


ON – Ontario


PE – Prince Edward Island


QC – Quebec


SK – Saskatchewan


Territories YT – Yukon Territory
NU – Nunavut

USA state abbreviations follow those of the US Postal Service.

### Discussion

Our knowledge of the diversity and distribution of Aleocharinae in Canada has increased rapidly over the last two decades (Klimaszewski et al. 2011, 2015a, Webster et al. 2016a, b). This increase may be attributed to a surge in sampling of this subfamily and intensive taxonomic studies, as well as the increased interest in aleocharines as a target group in forestry impact studies (Klimaszewski et al. 2008a, Pohl et al. 2007, 2008, Langor, unpublished data). Recently published contributions to the knowledge of aleocharine beetles in central Canada provided 33 new provincial records for the province of SK (Klimaszewski et al. 2015a). The present study, based on material from intensive collecting by D. Larson in southwestern SK provides 65 additional new records for the province and increased the number of known species there to 120. Of these 65 new provincial records, 12 represent species new to science, one record of an adventive species new to the province and North America (*Oligota inflata*), and 53 new provincial records of species known from other parts of Canada and or USA. It is interesting to note a high percentage of adventive species (16 sp., 13.3%), and a low number of Holarctic species (7 sp., 5.8%) in the SK fauna. The high percentage of adventive species is probably due to the highly modified prairie landscape that is responsible for supporting diverse habitats, and the inadequate knowledge of the total, very likely higher number of species. Agriculture has produced an environment unsuitable for many native species yet similar to European agricultural environments. Also, the sampling responsible for the species list presented here had a high bias towards habitats created in an active farm, habitats favoring synathropic species that are likely to be transported by man. The low number of Holarctic species is most likely due to poor collection in the north of the province. The Cypress Hills Upland is largely treed and contains a boreal element in its flora. However, much of its biota is derived from the western Cordillera thus contributing to the lower proportion of Holarctic species. From the 12 new species discovered, 8 represent Athetini (*Acrotona* - 1 sp., *Atheta* - 4 spp., *Dochmonota* - 3 spp.), one Aleocharini (*Aleochara* - 1 sp.), one Homalotini (*Agaricochara* - 1 sp.), one Oxypodini (*Oxypoda* - 1 sp.), and one Tachyusini (*Brachyusa* - 1 sp.). While new species in poorly known groups like *Acrotona*, *Agaricochara* and some subgenera of *Atheta*, are expected to increase with study efforts, it was surprising to see undescribed species in well studied genera like *Aleochara*, *Brachyusa* and *Oxypoda*. These species are from specialized habitats that were missed in collection or were not adequately sampled previously. The most interesting discoveries are 3 new species of native *Dochmonota* (Athetini), a genus previously know only from western Palaearctic with one species, *Dochmonota rudiventris* (Eppelsheim), recorded from eastern Canada as adventive, ID and MA (Klimaszewski et al. 2011, 2013b). Due to new distribution records (Bousquet et al. 2013), this is now considered a Holarctic species. The sampling effort by D. Larson in SK more than doubled the previously known species from the province, now standing at 122 species (Table 1).

In Canada, the Maritime provinces (NB, NS, NF, PE), and the YT are so far the best-studied regions of the country in terms of the aleocharine fauna (Klimaszewski et al. 2005, 2007b, 2008b, 2009a, b, 2010, 2011, 2012, Majka and Klimaszewski 2010, Webster et al. 2009, 2012, 2016a, b, Klimaszewski et al. 2015a). Some small areas of Quebec, Ontario, and coastal British Columbia have also received intensive sampling coupled with expert identification of material in recent years (Klimaszewski and Winchester 2002, Klimaszewski et al. 2007b, Brunke et al. 2012).

However, the large majority of central, western and northern Canada remains poorly studied. Large numbers of aleocharines (and other staphylinids) have been collected over the last 25 years as a result of numerous trapping studies in forests, native grasslands, agricultural lands, and wetlands, especially in Alberta (Klimaszewski et al. 2015a). The estimated underscribed/undiscovered aleocharine species in Canada was recently discussed in Klimaszewski et al. (2015a). Bousquet et al. (2013) recorded 27 species of aleocharines from SK, while Klimaszewski et al. (2015a) estimated that some additional 227 species are awaiting discovery in SK. In this paper we recognize 120 species in SK, so at least another 100 species may be awaiting discovery.

## New records and new species

### ALEOCHARINI Fleming

#### Aleochara (Echochara) elisabethae

Taxon classificationAnimaliaColeopteraStaphylinidae

Klimaszewski & Larson sp. n.

http://zoobank.org/6F4ECBB4-AA61-4E1F-A1D9-8CF0EBED4650

Figs 1–7

##### Holotype

(female). Canada, Saskatchewan, Bowie Ranch, 20 km NW Piapot, sand dunes, 29-V-2008, D. Larson (LFC). **Paratype**. Canada, Alberta, Empress, Alberta – Saskatchewan border, 5-VIII-1981, Lot 1, B.F. & J.L. Carr (CNC) 1 male.

##### Etymology.

This species is named for Dr. Élisabeth Gauthier, research director at LFC, for her continuous support of beetle biodiversity research in Canada.

##### Diagnosis.

**Figures 1–7.**
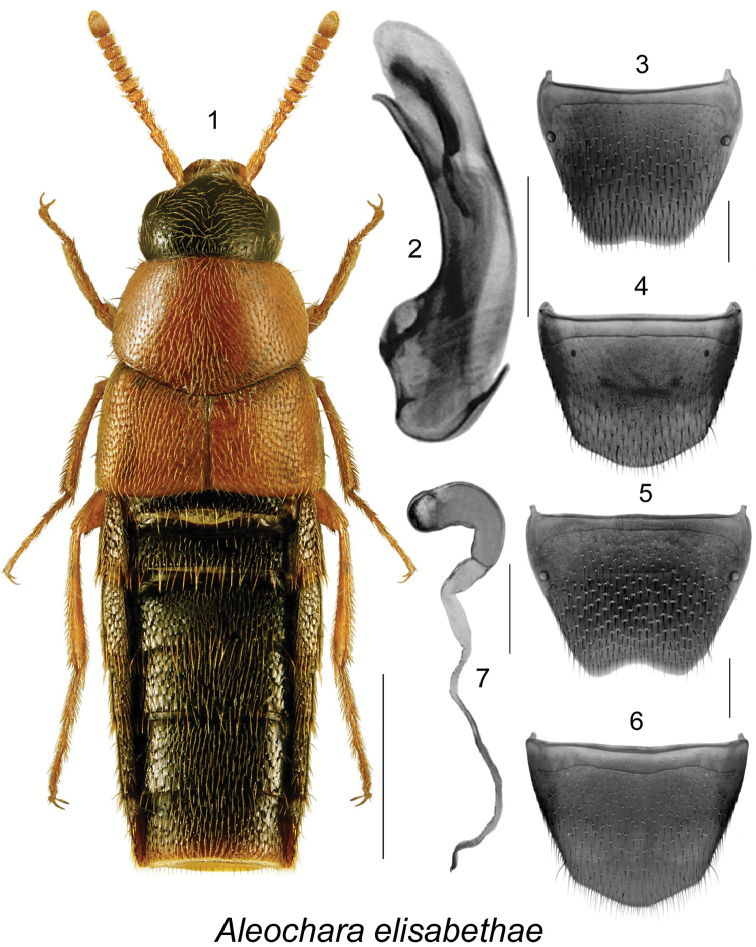
Aleochara (Echochara) elisabethae Klimaszewski & Larson, sp. n.: **1** habitus in dorsal view **2** median lobe of aedeagus in lateral view **3** male tergite VIII **4** male sternite VIII **5** female tergite VIII **6** female sternite VIII **7** spermatheca. Scale bar for habitus = 1 mm, and the remaining scale bars = 0.2 mm.

Body compact, narrowly oval (Fig. 1); head and abdomen dark brown, almost black, with pronotum, elytra and appendages orange (Fig. 1); length 3.8–4.3 mm; forebody with strong and dense meshed microsculpture; pubescence moderately dense; punctation coarser on eltra than elsewhere (Fig. 1); elytra at suture shorter than pronotum at middle length (Fig. 1); antennomeres V-X strongly transverse (Fig. 1); mesosternum not carinate. MALE. Tergite VIII shallowly emarginate apically (Fig. 3); sternite VIII rounded apically and slightly produced medially (Fig. 4); median lobe of aedeagus with tubus arcuate ventrally and with sharp apex, internal sac with elongate structures (Fig. 2). FEMALE. Tergite VIII emarginate apically (Fig. 5); sternite VIII rounded apically and slightly produced (Fig. 6); spermatheca with C-shaped tubular capsule, and short stem (Fig. 7).

This species is readily distingushed from remaining Nearctic species of subgenus Echochara by its strongly transverse and orange pronotum (dark brown or black in remaining species), arcuate tubus of median lobe of aedeagus with sharp apex (Fig. 2), and C-shaped swollen capsule of spermatheca (Fig. 7), which is narrower and club- or L-shaped in other species, and by the emarginated male and female tergite VIII (Figs 3, 5).

##### Distribution.

This species is known from the type localities in AB and SK.

##### Natural history.

The female holotype was captured on a dead ground squirrel in sand dunes. The male was collected in August from unspecified habitat. Species of subgenus Echochara are known from caves and animal burrows (Klimaszewski 1984).

#### Aleochara (Xenochara) inexpectata

Taxon classificationAnimaliaColeopteraStaphylinidae

Klimaszewski

(for diagnosis and illustrations, see Klimaszewski et al. 1984)

##### Distribution.

**Table:** 

Origin	Nearctic
Distribution	Canada: NB, NS, ON, QC, **SK**. USA: MI, WI
New provincial records	CANADA, **Saskatchewan**: Larson Ranch, Hwy 21, 16 km S Maple Creek, 20-X-2014, in dry polypore fungus, D. Larson (DLC) 1 female
References	Klimaszewski 1984, Webster et al. 2009, Brunke et al. 2012, Bousquet et al. 2013

##### Natural history.

In Saskatchewan, one female was captured in dry polypore fungus in October, and this constitutes the westernmost distribution record for this species. In NB, *Aleochara inexpectata* was collected from fresh moose dung in an eastern white cedar swamp and in decaying sea wrack resting on vegetation on the upper margin of a salt marsh. Adults were collected during May and June (Webster et al. 2009). Collection method: sifting.

#### Aleochara (Calochara) rubricalis

Taxon classificationAnimaliaColeopteraStaphylinidae

(Casey)

(for diagnosis and illustrations, see Klimaszewski et al. 1984)

##### Distribution.

**Table:** 

Origin	Nearctic
Distribution	Canada: BC, ON?, **SK**. USA: AZ, CA
New provincial records	CANADA, **Saskatchewan**: Larson Ranch, Hwy 21, 16 km S Maple Creek: 20-V-2008, D. Larson (LFC) 1 male; 25-VI-2008, carrion trap, D. Larson (DLC) 3 males, 1 female; 8-IV-2005, D. Larson (LFC) 1 female
References	Casey 1906, Klimaszewski 1984, Brunke et al. 2012 [one doubtfull record from ON], Bousquet et al. 2013

##### Natural history.

In Saskatchewan, specimens were collected from March through June, several adults were captured from carrion trap. Elsewhere, one specimen was taken from a mouse nest and other specimens were collected from February to October (Klimaszewski 1984).

#### Aleochara (Calochara) speculicollis

Taxon classificationAnimaliaColeopteraStaphylinidae

Bernhauer

(for diagnosis and illustrations, see Klimaszewski et al. 1984)

##### Distribution.

**Table:** 

Origin	Nearctic
Distribution	Canada: AB, ON, QC, **SK**. USA: CA, CO, AZ, MI, NV, TX
New provincial records	CANADA, **Saskatchewan**: Cypress Hills Park, Center Block, Sucker Creek, 1-4-VI-2012, D. Larson (LFC) 1 female.
References	Bernhauer 1901, Klimaszewski 1984, Bousquet et al. 2013

##### Natural history.

In Saskatchewan, one female was captured in June from unspecified habitat.

##### Comments.

We tentatively associate the SK specimen with this species because it is missing the spermatheca.

#### Aleochara (Coprochara) suffusa

Taxon classificationAnimaliaColeopteraStaphylinidae

(Casey)

(for diagnosis and illustrations, see Klimaszewski et al. 1984)

##### Distribution.

**Table:** 

Origin	Nearctic
Distribution	Canada: AB, BC, MB, QC, **SK**. USA: AK, AZ, CO, NM, WY
New provincial records	CANADA, **Saskatchewan**: Larson Ranch, Hwy 21, 16 km S Maple Creek: 27-V-2008, D. Larson (DLC) 1 female; 1-VI-2010, D. Larson (DLC, LFC) 2 females; 24-IX-2008, D. Larson (DLC) 1 female; 25-VI-2008, carrion trap, D. Larson (DLC) 1 female; 14-IX-2008, D. Larson (DLC) 1 sex undetermined; Cypress Lake, E dam, wind-drift, 9-V-2012, D. Larson (DLC) 1 female; Harris Res., 10 km S Maple Creek, 12-VI-2013, wind-drift, D. Larson (DLC) 1 male.
References	Casey 1906, Klimaszewski 1984, Bousquet et al. 2013

##### Natural history.

In Saskatchewan, one female was captured in a carrion trap and one from wind-drift. Elsewhere, specimens were found under rocks in a high altitude meadow and some from AB were reared in laboratory (Klimaszewski 1984).

##### Comments.

The SK specimens are darker and have only the central part of elytra reddish and the rest of the body piceous whereas the typical form of this species has the entire elytra orange or reddish-brown. Pubescence and punctation pattern and the genitalia of SK specimens are identical to the typical form with orange or reddish elytra.

#### Aleochara (Calochara) villosa

Taxon classificationAnimaliaColeopteraStaphylinidae

Mannerheim

(for diagnosis and illustrations, see Klimaszewski et al. 1984)

##### Distribution.

**Table:** 

Origin	Palaearctic, adventive in Canada
Distribution	Canada: AB, BC, NB, QC, **SK**. USA: AK, CA, OR, WA
New provincial records	CANADA, **Saskatchewan**: Larson Ranch, Hwy 21, 16 km S Maple Creek: 21-III-2007, sheep barn window, D. Larson (DLC) 2 males; 1-IV-2013, D. Larson (LFC) 1 female; 14-IV-2012, D. Larson (LFC) 1 male; 27-VII-2012, D. Larson (DLC) 1 female; 17-IX-2012, compost, D. Larson (DLC) 1 male.
References	Mannerheim 1830, Klimaszewski 1984, Webster et al. 2009, Bousquet et al. 2013

##### Natural history.

In SK, 2 males were captured from a sheep barn window, and one male was found in compost. SK specimens were collected in March, April, July and September. In New Brunswick, *Aleochara villosa* was collected from the nest contents of a great horned owl, *Bubo virginianus* (Gmelin) (Webster et al. 2009). Elsewhere, specimens have been collected from carrion and sifting an old hay pile (Klimaszewski 1984). Adults were collected in May. Collection method: sifting.

### ATHETINI Casey

#### Acrotona pseudopygmaea

Taxon classificationAnimaliaColeopteraStaphylinidae

Klimaszewski & Larson sp. n.

http://zoobank.org/E28F742F-730E-4D21-A43E-65FEEF229288

Figs 8–15

##### Holotype (male).

Canada, Saskatchewan, Larson Ranch, Hwy 21, 16 km S Maple Creek, 24-VII-2010, sifted from old mouldy alfalfa hay, D. Larson (LFC). **Paratypes**. 1 male and 1 female, with same label and collection data as the holotype (CNC).

##### Etymology.

The name of this species derives from the Latin participle *pygmaea*-, meaning small, and the prefix *pseudo*-, false. The genital structures of this species are similar to those of Palaearctic *Acrotona pygmaea* (Gravenhorst).

##### Diagnosis.

**Figures 8–15.**
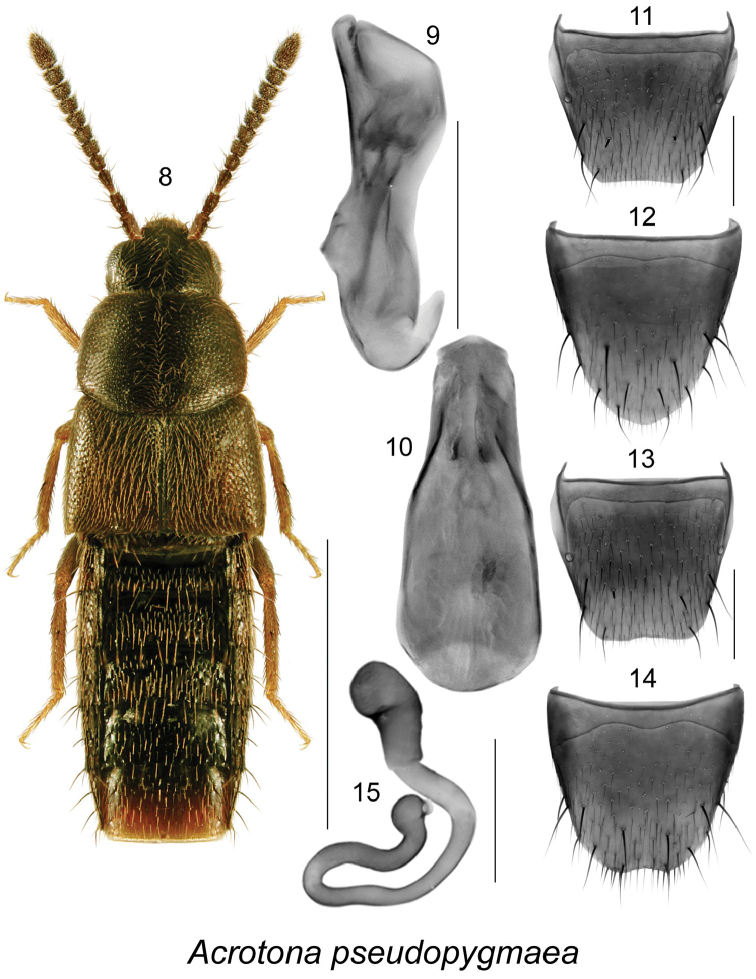
*Acrotona pseudopygmaea* Klimaszewski & Larson, sp. n.: **8** habitus in dorsal view **9** median lobe of aedeagus in lateral view, and **10** in dorsal view **11** male tergite VIII **12** male sternite VIII **13** female tergite VIII **14** female sternite VIII **15** spermatheca. Scale bar for habitus = 1 mm, and the remaining scale bars = 0.2 mm.

Body narrowly elongate, moderately convex, uniformly dark brown to almost black except for paler legs (Fig. 8); punctation on forebody fine, dense and asperate on elytra; head narrower than pronotum, ratio of maximum width of head to maximum width of pronotum 0.6; pronotum moderately transverse, ratio of maximum width to length 1.4, about as wide as elytra (Fig. 8); elytra at suture about as long as pronotum (Fig. 8); abdomen slightly narrowed posteriad; body length 2.4 mm; antennal articles V-X subquadrate. MALE. Tergite VIII moderately elongate and truncate apically (Fig. 11); sternite VIII rounded apically (Fig. 12); median lobe of aedeagus broad and rounded apically in dorsal view (Fig. 10), and tubus straight with apex facing upward in lateral view (Fig. 9). FEMALE. Tergite VIII truncate and slightly concave apically (Fig. 13); sternite VIII slightly emarginate apically (Fig. 14); spermatheca with tubular capsule and long, thin and sinuate posteriorly stem (Fig. 15).

Distinguished from all other *Acrotona* by the shape of median lobe of aedeagus with apex turned dorsally in lateral view (Fig. 9), by the shape of spermatheca with thin, long, sinuate, and posteriorly looped stem (Fig. 15), and by the shape of tergite and sternite VIII, which have basal margin straight and not sinuate (Figs 11–14).

##### Distribution.

This species is known only from the type locality in SK.

##### Natural history.

The type specimens were sifted from old mouldy alfalfa hay.

##### Comments.

This species is similar to Palaearctic *Acrotona pygmaea* (Gravenhorst) from which it differs by subquadrate antennal articles VI-X, by apex of tubus of median lobe of aedeagus more angular, female sternite VIII emarginated apically and spermatheca with much longer and broadly looped stem. It is also genitally similar to Nearctic *Acrotona actuella* (Casey) and *Acrotona egregiella* (Casey), from which it differs by straight and not sinuate ventral margin of tubus of median lobe of aedeagus, by straight and not sinuate basal margin of male tergite VIII, and by differently shaped spermatheca with posterior loop of stem sinuate.

#### Acrotona subpygmaea

Taxon classificationAnimaliaColeopteraStaphylinidae

(Bernhauer)

(for diagnosis and illustrations, see Webster et al. 2016b)

##### Distribution.

**Table:** 

Origin	Nearctic
Distribution	Canada: NB, NS, ON, **SK**
New provincial records	CANADA, **Saskatchewan**: Larson Ranch, Hwy 21, 16 km S Maple Creek, 5-6-VI-2013, maple litter, D. Larson (DLC) 1 female; 20-XI-2014, sifting willow leaf litter, D. Larson (DLC) 1 female.
References	Majka and Klimaszewski 2010, Brunke et al. 2012, Bousquet et al. 2013, Webster et al. 2016b

##### Natural history.

In SK, one female was captured from maple (*Acer negundo*) litter and one from willow (*Salix* spp.) leaf litter in June and October, respectively. In NB, *Acrotona subpygmaea* was found in litter of a variety of forest types and in wetlands including swamps, sphagnum bog, marshes and river margins. Specimens have also been taken from gilled mushroom and under bark (Webster et al. 2016b). Most adults were collected in May, with a few in April, June, August, and September.

#### Amischa analis

Taxon classificationAnimaliaColeopteraStaphylinidae

(Gravenhorst)

(for diagnosis and illustrations, see Klimaszewski et al. 2011)

##### Distribution.

**Table:** 

Origin	Palaearctic, adventive in Canada
Distribution	Canada: LB, NB, NF, NS, ON, QC, PE, **SK**
New provincial records	CANADA, **Saskatchewan**: Belanger Creek, Frenchman Valley, 18-X-2014, D. Larson (DLC) 1 female.
References	Moore and Legner 1975, Klimaszewski et al. 2005, Klimaszewski et al. 2007a, b, Majka and Klimaszewski 2010, Klimaszewski et al. 2011, Bousquet et al. 2013

##### Natural history.

In SK, one female was captured in October by sifting leaf litter along a creek. In NL, adults were collected in pitfall traps in agricultural fields, an urban field and on coastal sand dunes amidst vegetation, and the activity period was June to September (Klimaszewski et al. 2011). Elsewhere, adults in general occur in organic litter.

#### Atheta (Dimetrota) crenuliventris

Taxon classificationAnimaliaColeopteraStaphylinidae

Bernhauer

(for diagnosis and illustrations, see Klimaszewski et al. 2011)

##### Distribution.

**Table:** 

Origin	Nearctic
Distribution	Canada: LB, NB, NF, ON, QC, **SK**
New provincial records	CANADA, **Saskatchewan**, Larson Ranch, Hwy 21, 16 km S Maple Creek: 1-IX-2012, compost, D. Larson (DLC) 1 male; 8-IX-2012, compost, D. Larson (DLC) 1 male; Cypress Lake, E end, 31-VII-2012, sifting wrack, D. Larson (DLC) 1 female; Swift Current Cr., 28-VIII-2011, D. Larson (DLC) 1 female; Prince Albert, 53.9804, 106.2800, 532 m, 4-VI-2013, sand beach, sifting debris, B. Godin & D. Horwood (BGC, LFC) 2 males, 1 female.
References	Gusarov 2003, Lohse et al. 1990, Klimaszewski et al. 2005, Majka and Klimaszewski 2010, Bousquet et al. 2013

##### Natural history.

In SK, two males were found in compost in September, one female in wrack on lakeshore in July, and one female from unknown habitat in August. In NF, adults were collected from May to August in carrion-baited pitfall traps and flight intercept traps in conifer-dominated and mixedwood forests, and on the coastal barrens of southeastern LB (Klimaszewski et al. 2011). In NB, adults were collected in September from red spruce forest (Klimaszewski et al. 2005).

#### Atheta (Dimetrota) districta

Taxon classificationAnimaliaColeopteraStaphylinidae

Casey

(for diagnosis and illustrations, see Klimaszewski et al. 2011)

##### Distribution.

**Table:** 

Origin	Nearctic
Distribution	Canada: AB, BC, LB, NB, NF, NS, ON, QC, **SK**
New provincial records	CANADA, **Saskatchewan**, Cypress Hills Park, Center Block: Lodgepole Trail, 21-VIII-2013, dry and decaying mushrooms, D. Larson (DLC) 1 male; Highland Trail, 13-IX-2012, sifting spruce litter, D. Larson (DLC) 1 male.
References	Casey 1911, Klimaszewski et al. 2005, Majka and Klimaszewski 2008, 2010, Bousquet et al. 2013

##### Natural history.

In SK, one male was captured from dry and decaying mushroom, and another from spruce litter in September. In NF, adults were collected from June to August in carrion-baited pitfall traps and flight intercept traps in conifer-dominated and mixedwood forests, and on coastal barrens (Klimaszewski et al. 2011). In NB, adults were collected in June through September in red spruce forest (Klimaszewski et al. 2005).

#### Atheta (Dimetrota) pseudometlakatlana

Taxon classificationAnimaliaColeopteraStaphylinidae

Klimaszewski & Godin

(for diagnosis and illustrations, see Klimaszewski et al. 2008b)

##### Distribution.

**Table:** 

Origin	Nearctic
Distribution	Canada: **SK**, YT
New provincial records	CANADA, **Saskatchewan**, Cypress Hills Park, Center Block: Loch Lomond, 19-IX-2014, decaying mushrooms, D. Larson (DLC) 1 male, 1 female; 7-IX-2014, spruce-aspen, D. Larson (DLC) 1 female; fire guard, 10-IX-2013, decaying mushrooms, D. Larson (LFC) 1 male; Sucker Creek, 23-VI-204, aspen woodland, bracket gilled fungi, D. Larson (DLC) 1 male.
References	Klimaszewski et al. 2008b, Bousquet et al. 2013

##### Natural history.

In SK, specimens were collected from decaying mushrooms, bracket/gilled fungi, in spruce-aspen and aspen woodland forests. In YT adults were captured in June, July, and August at an elevation of 772 m in a white spruce and mixed white spruce-lodgepole pine forests (Klimaszewski et al. 2008b).

#### Atheta (Dimetrota) larsonae

Taxon classificationAnimaliaColeopteraStaphylinidae

Klimaszewski & Larson sp. n.

http://zoobank.org/4911C55F-055C-44C1-BE33-9ADE90B75144

Figs 16–20

##### Holotype (male).

Canada, Saskatchewan, Royal Edward Road, 25 km NW Maple Creek, 5-VI-2011, D. Larson (LFC).

##### Etymology.

The name of this species is dedicated to R.I. Larson. Ruby I. Larson was a geneticist at the Agriculture Canada Research Station, Lethbridge, who worked on wheat genetics. She was very active in promoting science and from 1958 to 1973 ran a Science Club for Junior High and High School age children. Members of this club went on to a variety of professional careers, including three (DJL included) who became professional entomologists. Her love of learning and science was infectious and her support and encouragement were major factors in our career choices. She taught us the joy and personal rewards of following one’s curiosity.

##### Diagnosis.

**Figures 16–20.**
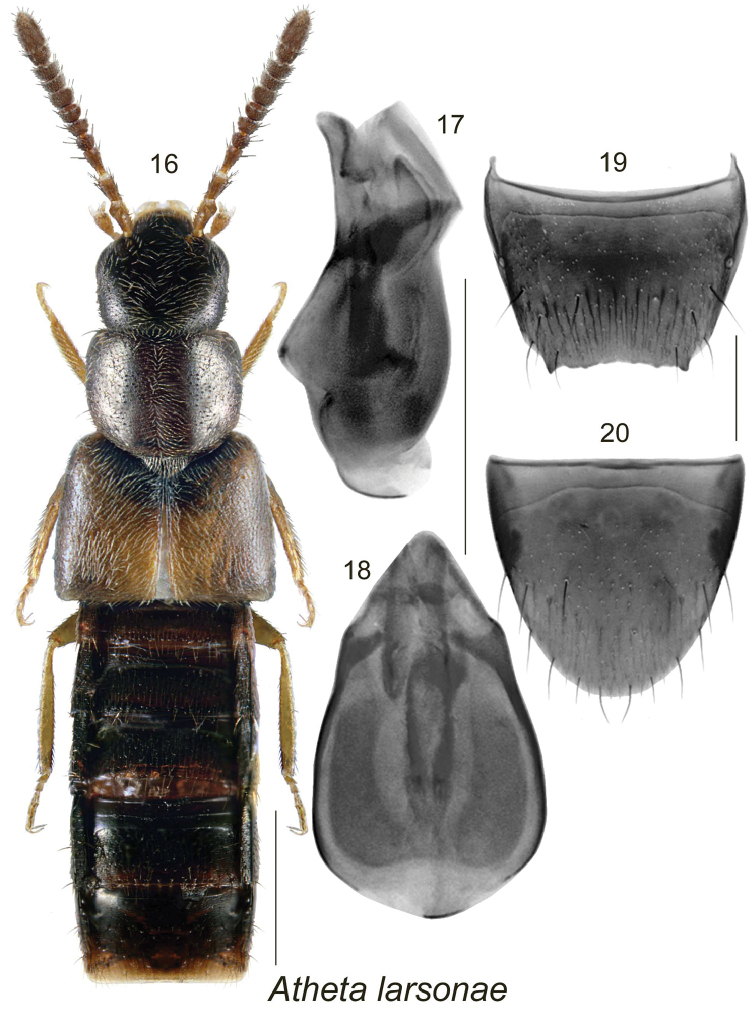
Atheta (Dimetrota) larsonae Klimaszewski & Larson, sp. n.: **16** habitus in dorsal view **17** median lobe of aedeagus in lateral view, and **18** in dorsal view **19** male tergite VIII **20** male sternite VIII. Scale bar for habitus = 1 mm, and the remaining scale bars = 0.2 mm.

Body narrowly elongate, slightly flattened (particularly on elytra), uniformly dark brown, almost black except for paler, light brown sutural section of elytra and legs (Fig. 16); punctation on forebody fine, dense and sparse; integument strongly glossy; head slightly narrower than pronotum; pronotum moderately transverse, and much narrower than elytra (Fig. 16); elytra strongly transverse, and at suture about as long as pronotum (Fig. 16); abdomen subparallel and distinctly narrower than elytra (Fig. 16); body length 2.5 mm; antennal articles V-X moderately transverse. MALE. Tergite VIII serrate apically with two larger lateral teeth (Fig. 19); sternite VIII rounded apically (Fig. 20); median lobe of aedeagus with broad and rounded bulbus and short and broadly triangular tubus in dorsal view (Fig. 18), and tubus straight with apex produced ventrally in lateral view (Fig. 17). FEMALE. Unknown.

Distinguished from all other Atheta (Dimetrota) by narrow head and pronotum, broad and short elytra, strongly glossy integument, and the shape of median lobe of aedeagus with apex produced ventrally in lateral view (Fig. 17).

##### Distribution.

This species is known only from the type locality in SK.

##### Natural history.

The holotype was captured in June from unspecified habitat.

##### Comments.

This species is superficially similar to Nearctic Atheta (Dimetrota) peticapensis Klimaszewski & Webster, with which it shares similar body proportions and enlarged bulbus of median lobe of aedeagus. However, these differences may not necessarily indicate a close relationship between these species.

#### Atheta (Dimetrota) strigosula

Taxon classificationAnimaliaColeopteraStaphylinidae

Casey

(for diagnosis and illustrations, see Klimaszewski et al. 2011)

##### Distribution.

**Table:** 

Origin	Nearctic
Distribution	Canada: BC, LB, NB, NF, ON, QC, **SK**, YT; USA: AK, NY
New provincial records	CANADA, **Saskatchewan**: Cypress Hills Park, Center Block, fire guard, 8-VIII-2013, gilled mushrooms, D. Larson (DLC) 1 male, 2 females; Lodgepole Trail, 21-VIII-2013, dry and decaying mushrooms, D. Larson (DLC) 1 female.
References	Casey 2010, Klimaszewski et al. 2005, 2008a, b, 2011, Bousquet et al. 2013

##### Natural history.

In SK, several females were found in dry and decaying mushrooms in August. In NF, adults were collected from June to October in carrion-baited and unbaited pitfall traps and in flight intercept traps in many forest types (coniferous, mixedwood and deciduous), and some adults were found in rotting mushrooms in forests (Klimaszewski et al. 2011). Elsewhere, adults were collected in June and August, from organic litter in red spruce forest in NB and forest litter in YT (Klimaszewski et al. 2005, 2008b).

#### Atheta (Dimetrota) terranovae

Taxon classificationAnimaliaColeopteraStaphylinidae

Klimaszewski & Langor

(for diagnosis and illustrations, see Klimaszewski et al. 2011, Brunke et al. 2012)

##### Distribution.

**Table:** 

Origin	Nearctic
Distribution	Canada: LB, NB, NF, ON, QC, **SK**, YT
New provincial records	CANADA, **Saskatchewan**: Cypress Hills Park, Center Block: fire guard, 18-VIII-2014, old polypore fungus on dead lodgepole pine stump, D. Larson (DLC) 2 males; 7-IX-2014, spruce-aspen, D. Larson (DLC) 1 male, 1 female.
References	Klimaszewski et al. 2011, Brunke et al. 2012, Klimaszewski et al. 2012, Webster et al. 2012, Bousquet et al. 2013

##### Natural history.

This species is frequently associated with forest mushrooms. In SK, specimens were captured from an old polypore fungus on dead lodgepole pine stump, and in spruce-aspen forest, in August and September. In NF, adults were collected from June to August in carrion-baited and unbaited pitfall traps and in flight intercept traps in many forest types (coniferous, mixedwood and deciduous), and some adults were found in rotting mushrooms in forests (Klimaszewski et al. 2011). In YT, specimens were found in mushrooms, in birch and mixed pine and willow forests, and white spruce and feathermoss forest in July and August (Klimaszewski et al. 2012). Most specimens from NB were collected from fresh and decaying gilled mushrooms. One individual was collected from a rotting lobster mushroom and another from a coral mushroom on a spruce log (Webster et al. 2012). This species was found in mixed forests, mature red spruce forests with red maple or birch, a black spruce forest, an eastern white cedar swamp, and a red oak forest (Webster et al. 2012). Adults from New Brunswick were collected during August, September (most specimens), and October (Webster et al. 2012).

#### Atheta (Microdota) pseudopittionii

Taxon classificationAnimaliaColeopteraStaphylinidae

Klimaszewski & Larson sp. n.

http://zoobank.org/9D833E80-70C3-4EBF-9ADC-5AFACB0D09BE

Figs 21–28

##### Holotype (male).

Canada, Saskatchewan, Larson Ranch, Hwy 21, 16 km S Maple Creek, 7-IX-2010, ex *Lepiota rhacodes*, D. Larson (LFC). **Paratypes**. Canada, Saskatchewan, Larson Ranch, Hwy 21, 16 km S Maple Creek: 25-VI-2008, carrion trap, D. Larson (CNC) 1 male, 1 female; 8-VII-2013, mushrooms, D. Larson (CNC, LFC) 1 male, 3 females; 15-VII-2014, decaying polypore mushroom, D. Larson (DLC, LFC) 2 females; 6-VIII-2013, ex *Lepiota rhacodes*, D. Larson (DLC, LFC) 3 males; 7-IX-2010, ex *Lepiota rhacodes*, D. Larson (DLC) 1 male, 2 females.

##### Etymology.

The species name *pseudopittionii* derived from the prefix *pseudo*- (false) and the specific name of European species *Atheta pittionii* Scheerpeltz, to which it is similar externally and has similar genitalia.

##### Diagnosis.

**Figures 21–28.**
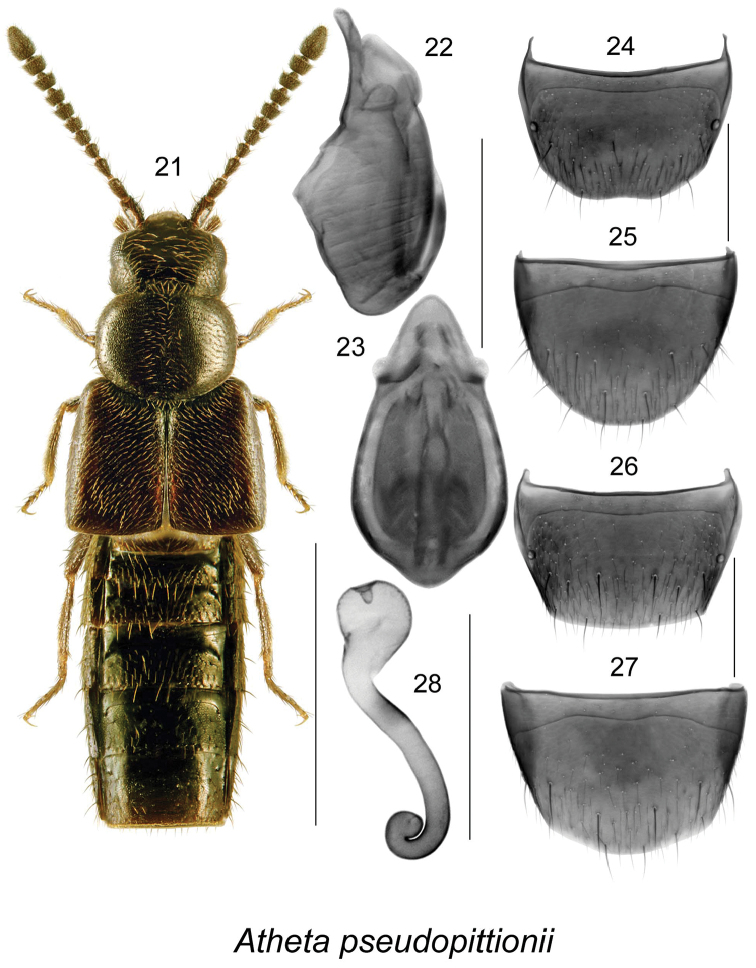
Atheta (Microdota) pseudopittionii Klimaszewski & Larson, sp. n.: **21** habitus in dorsal view **22** median lobe of aedeagus in lateral view, and **23** in dorsal view **24** male tergite VIII **25** male sternite VIII **26** female tergite VIII **27** female sternite VIII **28** spermatheca in lateral view;. Scale bar for habitus = 1 mm, and the remaining scale bars = 0.2 mm.

Body narrowly subparallel (Fig. 21), length 1.9-2.0 mm, uniformly black with tarsi yellowish; head, pronotum and elytra finely and sparsely punctate and pubescent, punctures small; integument strongly glossy, more so on abdomen, with meshed microsculpture; pronotum transverse, distinctly narrower than elytra, with pubescence directed obliquely anteriad anteriorly and obliquely posteriad posteriorly from median line of disc (Fig. 21); elytra at suture distinctly longer than pronotum (Fig. 21); abdomen subparallel. MALE. Tergite VIII truncate apically and slightly emarginate (Fig. 24); sternite VIII rounded apically (Fig. 25). Median lobe of aedeagus with large oval bulbus, and short and broadly triangular tubus in dorsal view (Fig. 23), in lateral view tubus arcuate with base near bulbus sinuate (Fig. 22); internal sac structures as illustrated (Figs 22, 23). FEMALE. Tergite VIII truncate apically (Fig. 26); sternite VIII broadly arcuate apically (Fig. 27); spermatheca with spherical capsule bearing narrow apical invagination, stem narrow, and with a small coiled apex (Fig. 28).

This species is very similar to European *Atheta pittionii* Scheerpeltz, from which it differs by broader and more elongate elytra, larger bulbus of median lobe of aedeagus in dorsal view (Fig. 23), more sinuate base of tubus of median lobe of aedeagus in lateral view (Fig. 22), and differently shaped complex structures of the internal sac (Figs 22, 23). For genitalia of *Atheta pittionii*, see Brundin (1948) [under the name of *Atheta parvicornis*].

##### Distribution.

Adults are known only from SK.

##### Natural history.

Most adults of this species were collected from Shaggy parasol mushrooms, *Chlorophyllum rhacodes* (=*Lepiota rhacodes*), from unspecified mushrooms, and from carrion.

#### Atheta (Microdota) riparia

Taxon classificationAnimaliaColeopteraStaphylinidae

Klimaszewski & Godin

(for details and body image, see Klimaszewski et al. 2012)

##### Distribution.

**Table:** 

Origin	Nearctic
Distribution	Canada: **SK**, YT
New provincial records	CANADA, **Saskatchewan**: Cypress Hills Park, Center Block: fire guard, Sucker Creek, 23-VI-2014, aspen woodland bracket/gilled fungi, D. Larson (DLC) 1 male; 7-IX-2014, spruce-aspen, D. Larson (DLC) 1 male.
References	Klimaszewski et al. 2012, Bousquet et al. 2013

##### Natural history.

One SK male was captured in bracket/gilled fungi in aspen woodland in June, and the other from spruce-aspen woodland in September. In YT, two males were captured by sifting litter in mixed aspen and white spruce forest in September, and one female was found on a mushroom in August (Klimaszewski et al. 2012).

#### Atheta (Microdota) spermathecorum

Taxon classificationAnimaliaColeopteraStaphylinidae

Klimaszewski & Larson sp. n.

http://zoobank.org/8561AFDD-2420-4FC5-8B6D-F1AED26157B7

Figs 29–32

##### Holotype

(female). Canada, Saskatchewan, Larson Ranch, Hwy 21, 16 km S Maple Creek, 8-VI-2014, D. Larson (LFC). **Paratypes**. Canada, Saskatchewan, Larson Ranch, Hwy 21, 16 km S Maple Creek: 29-V-2012 (LFC) 1 female; 30-V-2014, D. Larson (CNC) 1 female; 17-VII-2014, decaying polypore mushroom, D. Larson (CNC) 1 female; Belanger Creek, Frenchman Valley, 11-V-2013, D. Larson (DLC) 1 female; Harris Res., 10 km S Maple Creek, 20-V-2004, drift, D. Larson (DLC) 1 female; Alberta, Lethbridge, 24-III-1964, D. Larson (DLC) 1 female.

##### Etymology.

The species name *spermathecorum* is derived from the name of spermatheca in reference to unusually shaped capsule of the spermatheca of this species.

##### Diagnosis.

**Figures 29–32.**
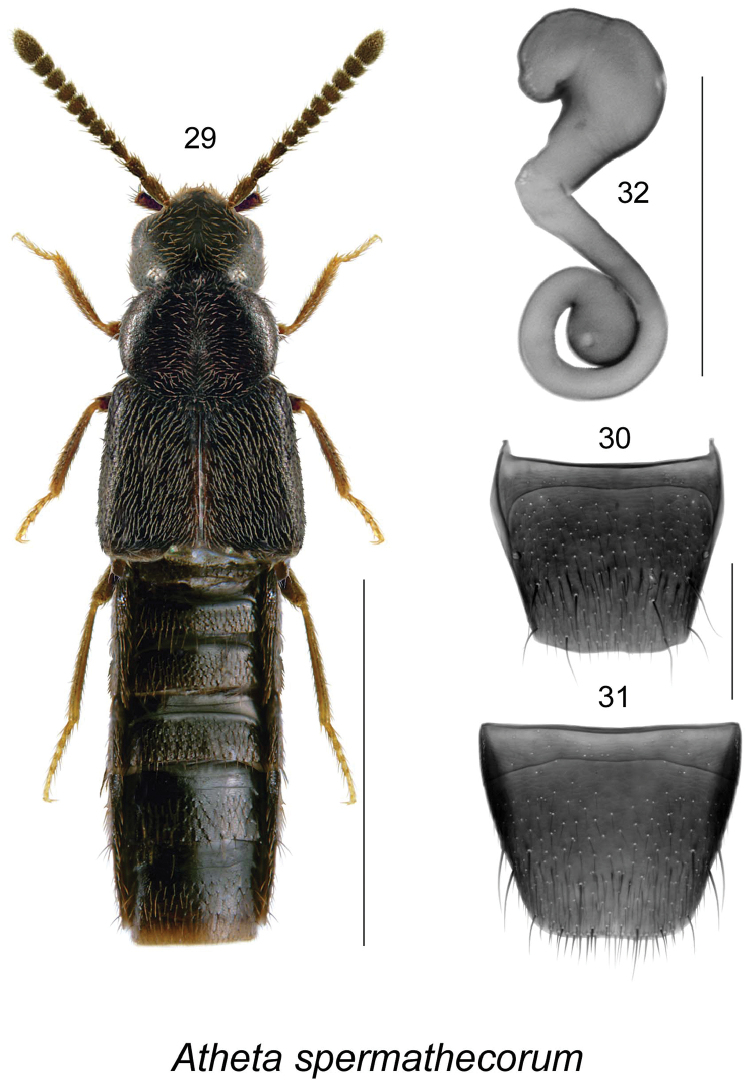
Atheta (Microdota) spermathecorum Klimaszewski & Larson, sp. n. (female): **29** habitus in dorsal view **30** tergite VIII **31** sternite VIII **32** spermatheca. Scale bar for habitus = 1 mm, and the remaining scale bars = 0.2 mm.

Body narrowly subparallel (Fig. 29), length 1.9-2.2 mm, uniformly black, legs with at least tarsi reddish-brown; head, pronotum and elytra finely and moderately densely punctate and pubescent, punctures small (Fig. 29); integument moderately glossy, more so on abdomen; pronotum transverse, narrower than elytra, with pubescence directed obliquely anteriad and posteriad posteriorly from median line of disc (Fig. 29); elytra at suture slightly longer than pronotum; abdomen subparallel (Fig. 29). MALE. Unknown. FEMALE. Tergite VIII truncate and slightly concave apically (Fig. 30); sternite VIII truncate and slightly emarginate apically (Fig. 31); spermatheca with irregularly-shaped capsule without apparent apical invagination, stem narrow, and with a single posterior coil bearing swollen apical part (Fig. 32).

It is distinguished from all other Nearctic species of *Atheta*, subgenus Microdota, by the unique shape of spermatheca bearing bulbus apical projection on top of capsule (Fig. 32).

##### Distribution.

Adults are known from SK and AB.

##### Natural history.

Females were captured in March (Alberta), May and July (Saskatchewan): one was found in a decaying polypore mushroom and one was found in lake drift in May.

#### Atheta (Rhagocneme) subsinuata

Taxon classificationAnimaliaColeopteraStaphylinidae

Erichson

(for details and body image, see Klimaszewski et al. 2008b)

##### Distribution.

**Table:** 

Origin	Palaearctic, adventive in Canada
Distribution	Canada: **SK** , YT
New provincial records	CANADA, **Saskatchewan**, Larson Ranch, Hwy 21, 16 km S Maple Creek, 1-VI-2011, D. Larson (DLC) 1 female; 27-VI-2010, old wet alfalfa hay with *Coprinus*, D. Larson (DLC, LFC) 3 females; 24-VII-2010, sifted from old mouldy alfalfa hay, D. Larson (DLC) 1 female; 1-IX-2012, compost, D. Larson (LFC) 1 female.
References	Klimaszewski et al. 2008b, Bousquet et al. 2013

##### Natural history.

Like many introduced species, *Atheta subsinuata* appears to be synanthropic, as all collections have been made from artificial habitats. The Saskatchewan specimens were sifted from old mouldy alfalfa hay in June and July, and one female was taken in September from compost. In YT, four specimens were captured in a compost pile in September 2005 (Klimaszewski et al. 2008b).

#### Atheta (Tetropla) frosti

Taxon classificationAnimaliaColeopteraStaphylinidae

Bernhauer

(for details and illustrations, see Gusarov 2003, Klimaszewski et al. 2011)

##### Distribution.

**Table:** 

Origin	Nearctic
Distribution	Canada: BC, LB, NB, NS, ON, QC, **SK**; USA: MA, NC, NH, NY, PA, RI, VT
New provincial records	CANADA, **Saskatchewan**, Cypress Hills, Center Block, Lake, Lodgepole Trail, 24-IX-2014, decaying mushrooms, D. Larson (DLC) 1 female.
References	Gusarov 2003, Klimaszewski et al. 2005, Majka and Klimaszewski 2008, 2010, Klimaszewski et al. 2011, Bousquet et al. 2013

##### Natural history.

The SK female was captured in decaying mushrooms in September. In LB, adults were abundant in pitfall traps during July and August in an open spruce forest with sandy soil and *Cladina* lichen cover, and a few adults were captured using pitfall traps in a birch-dominated forest (Klimaszewski et al. 2011). Elsewhere, adults occurred from July to October in organic debris in red spruce forest, in polypore fungus in coniferous forest, and on the forest floor in red oak and deciduous forests (Klimaszewski et al. 2005, Majka and Klimaszewski 2008, 2010).

### Incertae sedis

The following species have uncertain subgeneric affiliation in the large and diverse genus *Atheta*. Some of the species belong to a group of species described in Europe by Benick and Lohse (1974) as the “Mischgruppe” (mixed group) of *Atheta*.

#### Atheta pseudoschistoglossa

Taxon classificationAnimaliaColeopteraStaphylinidae

Klimaszewski & Webster

(for details, genitalia and body image, see Webster et al. 2016b)

##### Distribution.

**Table:** 

Origin	Nearctic
Distribution	Canada: NB, **SK**
New provincial records	CANADA, **Saskatchewan**: Cypress Hills Park, Sucker Creek, 21-VIII-2012, aspen-pine litter, D. Larson (DLC) 1 male; Cypress Hills Park, Center Block, Lodgepole Trail, 18-IX-2012, pine-spruce litter near stream, D. Larson (DLC) 1 female; Belanger Creek, Frenchman Valley, 18-X-2014, D. Larson (DLC) 1 male.
References	Webster et al. 2016b

##### Natural history.

The SK specimens were captured from aspen/pine litter and pine/spruce litter in August through October. In NB, most adults of *Atheta pseudoschistoglossa* were found in or near wetland habitats including among cobblestones, drift material, and flood debris along river margins, moist leaves along vernal pond margin in a silver maple swamp, in leaf litter and moss along brook margins in alder swamps, and in litter at base of red maple, in *Carex* hummock in *Carex* marshes, in leaf litter in a red oak forest near seasonally flooded marsh, in a salt marsh, in marsh litter in a *Carex*-sedge marsh, and in litter and sphagnum at the base of a tree in a marsh (Webster et al. 2016b). A few adults were captured in Lindgren funnel traps in hardwood woodland near a seasonally flooded marsh and in an old mixed forest (Webster et al. 2016b). Adults were collected from mid-April to August (Webster et al. 2016b).

#### Atheta remulsa

Taxon classificationAnimaliaColeopteraStaphylinidae

Casey

(for details and illustrations, see Klimaszewski et al. 2011)

##### Distribution.

**Table:** 

Origin	Nearctic
Distribution	Canada: AB, BC, LB, NB, NF, NS, ON, QC, **SK**, YT
New provincial records	CANADA, **Saskatchewan**: Cypress Hills Park, Center Block, Lodgepole Trail, 21-VIII-2013, dry and decaying mushrooms, D. Larson (DLC) 1 female.
References	Casey 1910, Klimaszewski et al. 2005, 2007b, Majka and Klimaszewski 2008, 2010, Bousquet et al. 2013

##### Natural history.

In SK one female was captured from dry and decaying mushrooms. In NL, adults were collected from June to September using unbaited and carrion-baited pitfall traps and flight intercept traps in many forest types (deciduous, mixedwood, coniferous, riparian), and also in rotting mushrooms in forests (Klimaszewski et al. 2011). Elsewhere, adults were collected in NB from red spruce mixed forest from June through September (Klimaszewski et al. 2005), and in QC from yellow birch/balsam fir dominated forest in June and July (Klimaszewski et al. 2007b).

#### Atheta richardsoni

Taxon classificationAnimaliaColeopteraStaphylinidae

Klimaszewski & Larson sp. n.

http://zoobank.org/D56426E0-874E-4E33-B620-5DB5EFA42097

Figs 33–40

##### Holotype (male).

Canada, Saskatchewan, Hwy 21, 20 km N Maple Creek, 25-VI-2010, Gramma-stipa pasture, Richardson ground squirrel burrow, D. Larson (LFC). Paratype. Canada, Saskatchewan, Grassland National Park, W Block Larson’s Prairie Dog colony, 11-VI-2009, D. Larson (LFC) 1 female.

##### Etymology.

This species name is derived from the surname of Sir John Richardson, the surgeon-naturalist who participated in 19th century British naval expeditions to the arctic coast of “British North America”, now Canada. In 1820 he discovered a new species of ground squirrel along the Saskatchewan River, which was later named after him as *Urocitellus richardsonii*. The holotype of *Atheta richardsoni* was found in a Richardson’s ground squirrel burrow.

##### Diagnosis.

**Figures 33–40.**
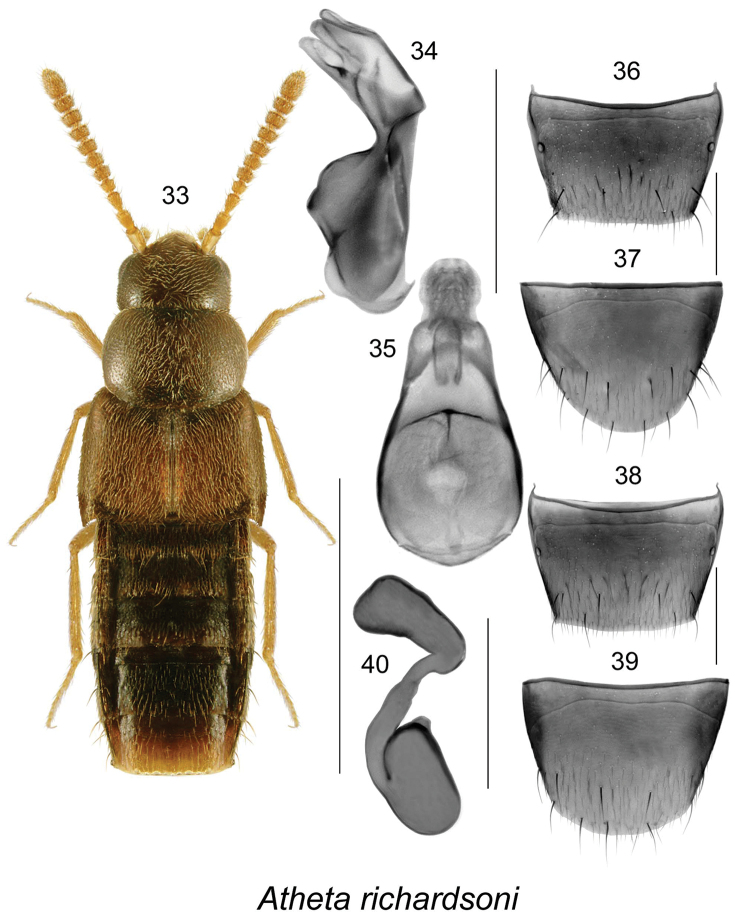
*Atheta* (*sensu lato*) *richardsoni* Klimaszewski & Larson, sp. n.: **33** habitus in dorsal view **34** median lobe of aedeagus in lateral view, and **35** in dorsal view **36** tergite VIII **37** sternite VIII **38** female tergite VIII **39** female sternite VIII **40** spermatheca. Scale bar for habitus = 1 mm, and the remaining scale bars = 0.2 mm.

Body narrowly subparallel (Fig. 33), length 1.9 mm, dark brown, with appendages yellowish-brown; head, pronotum and elytra finely and densely punctate and pubescent, punctures small, all pubescence directed straight or obliquely posteriad; integument moderately glossy, more so on abdomen (Fig. 33); pronotum transverse, narrower than elytra, with pubescence directed straight posteriad on median line of disc (Fig. 33); elytra at suture about as long as pronotum (Fig. 33); abdomen subparallel. MALE. Tergite VIII truncate apically (Fig. 36); sternite VIII broadly rounded apically (Fig. 37). Median lobe of aedeagus with large oval bulbus and broad tubus rapidly tapering near apex in dorsal view (Fig. 35), in lateral view tubus straight and narrowly rounded at apex, strongly produced ventrally (Fig. 34); internal sac structures as illustrated (Figs 34, 35). FEMALE. Tergite VIII transverse and truncate apically (Fig. 38); sternite VIII broadly arcuate apically, antecostal suture strongly sinuate (Fig. 39); spermatheca with narrowly pitcher-shaped capsule and thin stem ending with enlarged, sac-like posterior part (Fig. 40).

Distinguished from all other species of Nearctic *Atheta* by its small size, densely and finally punctate and pubescent forebody, nearly all pronotal pubescence directed straight posteriad (Fig. 33), the shape of median lobe of aedeagus with very broad tubus of median lobe in dorsal view (Fig. 35), and the shape of spermatheca with enlarged, sac-shaped posterior part of stem (Fig. 40).

##### Distribution.

Adults are known from SK.

##### Natural history.

The single male from SK was captured in a ground squirrel burrow, and the single female was found in a Prairie Dog colony in June.

##### Comments.

This species in body size and general appearance is similar to species of the subgenus Microdota of *Atheta*. However, it has a different pubescence pattern of pronotum with microsetae along midline of disc directed straight posteriad and elsewhere straight or obliquely posteriad (Fig. 33), and pubescence on elytra with microsetae directed approximately straight posteriad (Fig. 33). The tubus of the median lobe of the aedeagus is very broad and abruptly narrowed apically in dorsal view (Fig. 34), and spermatheca has enlarged and sac-shaped posterior part of stem (Fig. 40). These are unique features of this species, which slightly resemble those of European *Atheta liturata* Stephens, which has a similarly shaped median lobe of aedeagus and spermatheca, but the European species has a differently shaped male tergite VIII with strong lateral projections (for illustrations, see Palm 1970). The European species is known from mushrooms. Benick and Lohse (1974) assigned *Atheta liturata* to *Atheta* (Mischgruppe III, IV).

#### Dinaraea angustula

Taxon classificationAnimaliaColeopteraStaphylinidae

(Gyllenhal)

(for details and illustrations, see Klimaszewski et al. 2011, 2013a, b)

##### Distribution.

**Table:** 

Origin	Palaearctic, adventive in Canada
Distribution	Canada: AB, LB, NB, NF, NS, ON, PE, QC, **SK**, YT. USA: CA, NY
New provincial records	CANADA, **Saskatchewan**: Saskatoon, 16-VI-1976, D. Larson (DLC) 1 female; Larson Ranch, Hwy 21, 16 km S Maple Creek, 5-V-2008, D. Larson (DLC, LFC) 2 males; 22-VI-2014, D. Larson (LFC) 1 male.
References	Moore and Legner 1975, Muona 1984, Klimaszewski et al. 2007a, Webster et al. 2009, Majka and Klimaszewski 2010, Klimaszewski et al. 2011, 2013a, b, Bousquet et al. 2013

##### Natural history.

The SK specimens were captured in May and June from unspecified habitat. Elsewhere, this species is associated with soil and organic debris in agricultural fields and disturbed urban meadows. It is also found in marsh litter, in leaf litter in mixed forests, in compost, under bark of decaying spruce logs, amongst vegetation on a coastal sand dune, in litter in a cattail marsh, in leaf litter along a vernal pond, and in drift material along a lakeshore (Webster et al. 2009, Klimaszewski et al. 2010, 2011, 2013a, b). The adult activity period is April to September.

#### Dinaraea pacei

Taxon classificationAnimaliaColeopteraStaphylinidae

Klimaszewski & Langor

(for details and illustrations, see Klimaszewski et al. 2011, 2013a)

##### Distribution.

**Table:** 

Origin	Nearctic
Distribution	Canada: AB, BC, LB, NB, ON, PE, QC, **SK**, YT. USA: AK
New provincial records	CANADA, **Saskatchewan**: Cypress Hills Park, Center Block: fire, Sucker Creek, 23-VI-2014, aspen woodland bracket/gilled fungi, D. Larson (DLC) 1 female; 1-VI-2004, under aspen bark, Hooper & Larson (DLC) 1 male.
References	Webster et al. 2009, Majka and Klimaszewski 2010, Klimaszewski et al. 2011, 2013a, Bousquet et al. 2013

##### Natural history.

The SK specimens were captured from aspen woodland bracket/gilled fungi, and from under aspen bark. Adults in NF and LB were collected from June to August using pitfall traps and flight intercept traps in various coniferous forest types, and one specimen was collected under the bark of a dead red pine (Klimaszewski et al. 2011). In BC, adults were caught in July and September in emergence traps attached to the trunks of lodgepole pine (*Pinus contorta* Dougl. ex Loud. *latifolia* Engelm.) infested by mountain pine beetle (*Dendroctonus ponderosae* Hopkins) (Klimaszewski et al. 2013a). In NB, adults were found: under the bark of large fallen spruce in an old-growth eastern white cedar swamp; under tight bark of American elm; in a silver maple forest; in fleshy polypore fungi at the base of a dead standing *Populus* sp. in a wet alder swamp; and in a group of *Pholiota* sp. at the base of a dead *Populus* sp. in a mixed forest. In Quebec, adults were found in dead black spruce in a black spruce forest (Webster et al. 2009). Adults were also captured in Lindgren funnel traps deployed in an old-growth white spruce (*Picea glauca* (Moench) Voss) and balsam fir forest, an old mixed forest with red and white spruce, red and white pine (*Pinus strobus* L.), and a rich Appalachian hardwood forest with some conifers (Webster et al. 2009). Adults were collected from March to September (Webster et al. 2009).

#### Dochmonota

Taxon classificationAnimaliaColeopteraStaphylinidae

Thomson

(for synonymies and discussion, see Gusarov 2003)

##### Remark.

Untill now, only one native species, *Dochmonota rudiventris* (Eppelsheim) (Figs 41–48), was reported from North America including Canada (Gusarov 2003, Klimaszewski et al. 2011).

**Figures 41–48.**
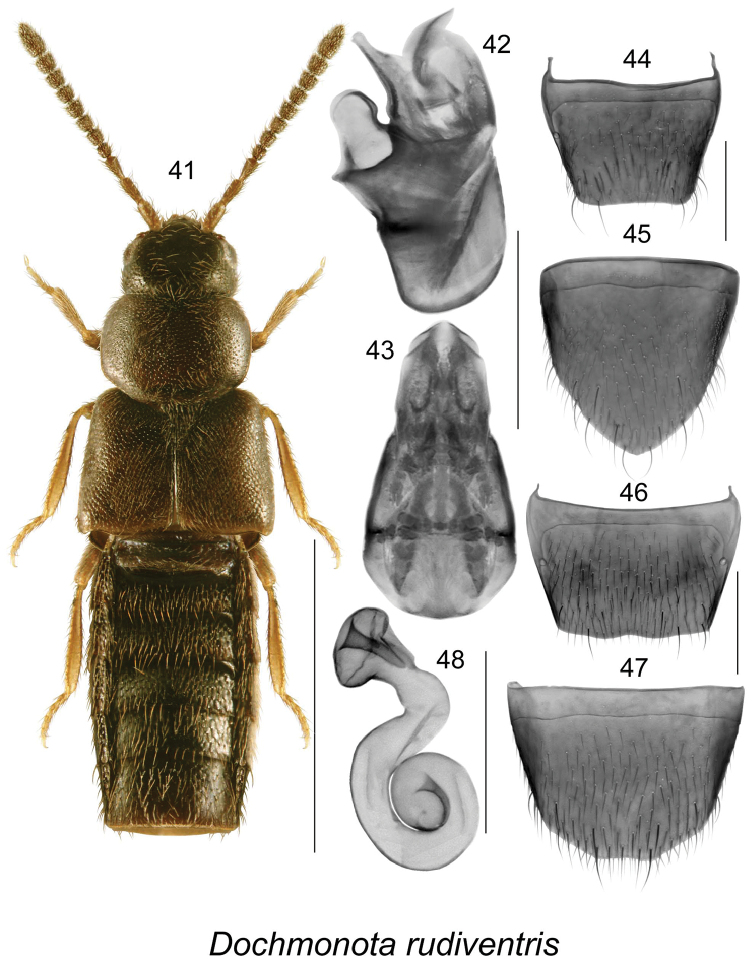
*Dochmonota rudiventris* (Eppelsheim): **41** habitus in dorsal view **42** median lobe of aedeagus in lateral view, and **43** in dorsal view **44** male tergite VIII **45** male sternite VIII **46** female tergite VIII **47** female sternite VIII **48** spermatheca. Scale bar for habitus = 1 mm, and the remaining scale bars = 0.2 mm.

##### Key to Nearctic species of *Dochmonota*

**Table:** 

1	Head about as broad as pronotum (Fig. 49); body narrow with elytra at base only slightly broader than pronotum (Fig. 49); male sternite VIII notched dorsally (Fig. 53); ventral margin of tubus of median lobe of aedeagus straight with base slightly sinuate in lateral view (Fig. 50); spermatheca with capsule broad, pitcher-shaped, and stem coiled (Fig. 56)	***Dochmonota langori* Klimaszewski & Larson, sp. n.**
–	Head distinctly narrower than pronotum (Figs 41, 57, 65); body broad with elytra at base distinctly broader than pronotum (Figs 41, 57, 65); male sternite VIII with apex entire (Figs 45, 61, 69); ventral margin of tubus of median lobe of aedagus diffferently shaped (Figs 42, 58, 66); spermatheca with capsule moderately broad, subspherical and stem coiled (Figs 48, 64, 72)	2
2	Elytra at suture longer than pronotum (Fig. 57); male tergite VIII with two small lateral teeth at the apical margin (Fig. 60); median lobe of aedeagus with sinuate apical margin of tubus (Fig. 58); spermatheca with capsule subspherical and with twisted stem (Fig. 64)	***Dochmonota simulans* Klimaszewski & Larson, sp. n.**
–	Elytra at suture about as long as pronotum (Figs 41, 65); male tergite VIII without teeth on apical margin (Figs 44, 68); median lobe of aedeagus with straight apical margin of tubus in lateral view (Figs 42, 66)	3
3.	Elytral posterior corners with strong lateral emarginations (Fig. 41); median lobe of aedeagus with large crista apicalis of bulbus (Fig. 42); spermatheca with capsule compressed dorso-ventrally (Fig. 48)	***Dochmonota rudiventris* (Eppelsheim)**
–	Elytral posterior corners with slight emarginations laterally (Fig. 65); median lobe of aedeagus with small crista apicalis of bulbus (Fig. 66); spermatheca with capsule spherical (Fig. 72)	***Dochmonota websteri* Klimaszewski & Larson, sp. n.**

**Figures 49–56.**
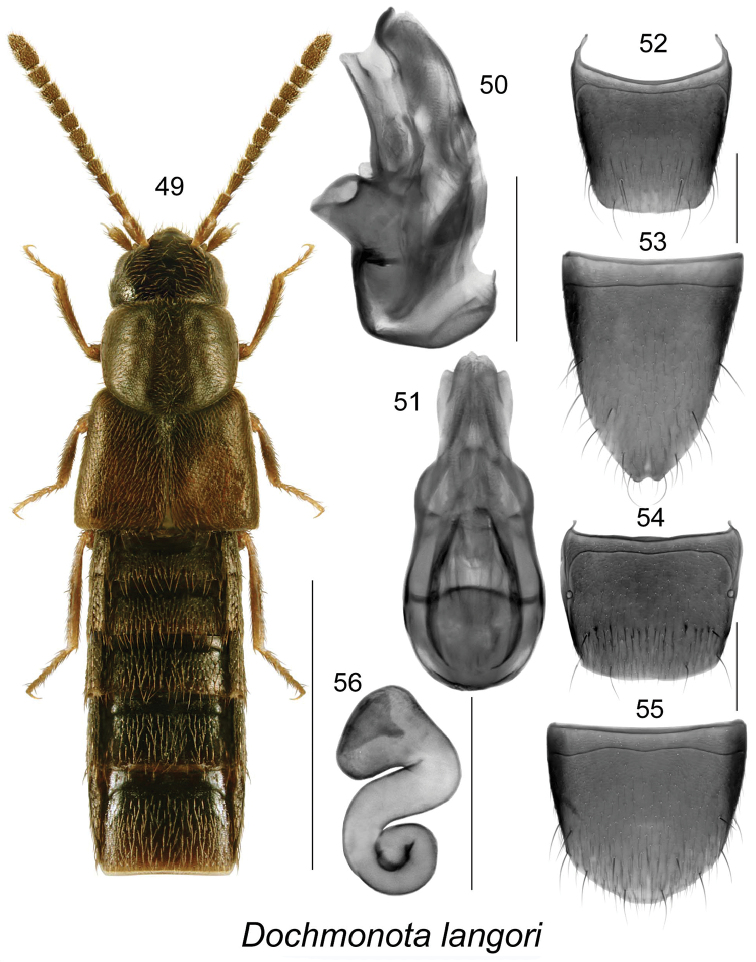
*Dochmonota langori* Klimaszewski & Larson, sp. n.: **49** habitus in dorsal view **50** median lobe of aedeagus in lateral view, and **51** in dorsal view **52** male tergite VIII **53** male sternite VIII **54** female tergite VIII **55** female sternite VIII **56** spermatheca. Scale bar for habitus = 1 mm, and the remaining scale bars = 0.2 mm.

**Figures 57–64.**
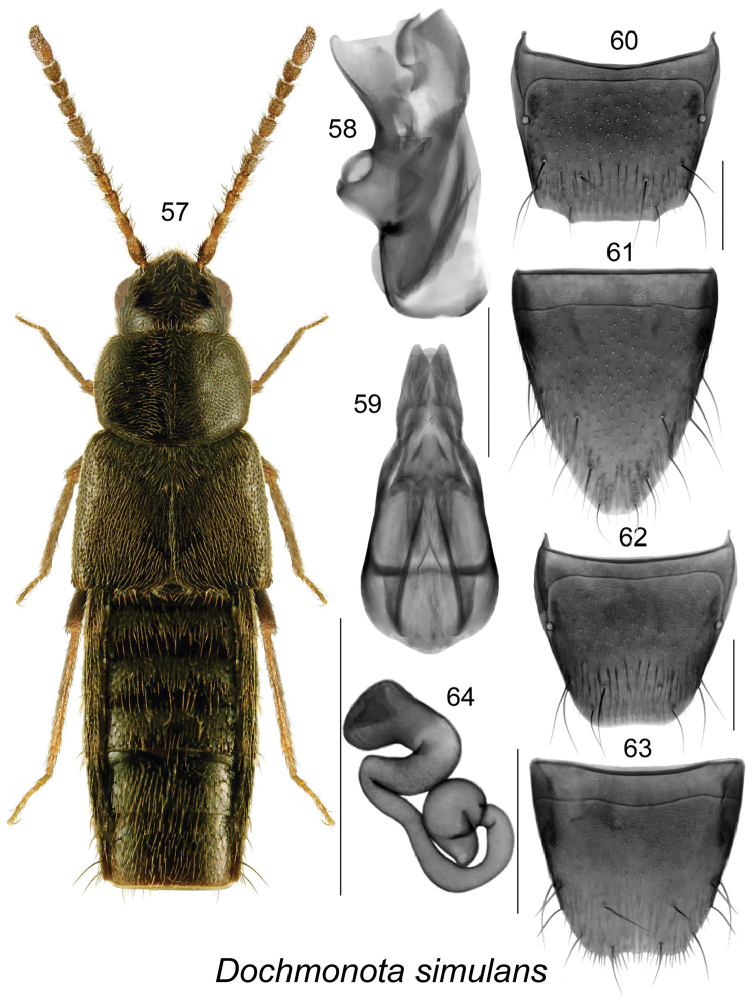
*Dochmonota simulans* Klimaszewski & Larson, sp. n.: **57** habitus in dorsal view **58** median lobe of aedeagus in lateral view, and **59** in dorsal view **60** male tergite VIII **61** male sternite VIII **62** female tergite VIII **63** female sternite VIII **64** spermatheca. Scale bar for habitus = 1 mm, and the remaining scale bars = 0.2 mm.

**Figures 65–72.**
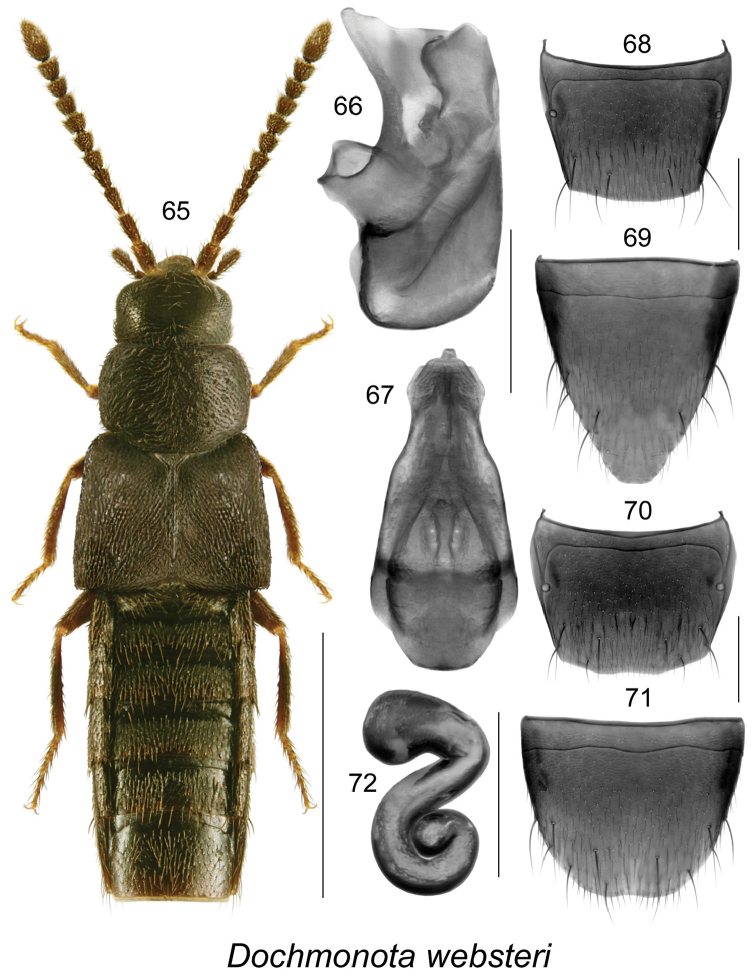
*Dochmonota websteri* Klimaszewski & Larson, sp. n.: **65** habitus in dorsal view **66** median lobe of aedeagus in lateral view, and **67** in dorsal view **68** male tergite VIII **69** male sternite VIII **70** female tergite VIII **71** female sternite VIII **72** spermatheca. Scale bar for habitus = 1 mm, and the remaining scale bars = 0.2 mm.

#### Dochmonota langori

Taxon classificationAnimaliaColeopteraStaphylinidae

Klimaszewski & Larson sp. n.

http://zoobank.org/60D5577B-AD81-414F-A167-5E8375999138

Figs 49–56

##### Holotype (male).

Canada, Saskatchewan, Cypress Lake, E dam, 9-V-2012, wind-drift, D. Larson (LFC) 1 male. **Paratypes**. Canada, Saskatchewan, Cypress Lake, E dam, 9-V-2012, wind-drift, D. Larson (CNC, LFC) 3 females; Cypress Lake, E dam, 31-VII-2012, sifting wrack, D. Larson (DLC) 2 females; Crane Lake, NE Piapot, 28-VIII-2011, beach wrack, D. Larson (CNC) 1 female.

##### Etymology.

The species is named for our friend and professional colleague Dr. David W. Langor, Canadian Forest Service, collaborator and supporter of many joint entomological projects. He contributed to the discovery and descriptions of many new species of aleocharine beetles in Canada, particularly in Newfoundland and Alberta.

##### Diagnosis.

Body narrowly subparallel (Fig. 49), length 2.2-2.5 mm, uniformly black; head, pronotum and elytra finely and densely punctate, punctures small; pubescence dense; integument moderately glossy, more so on abdomen, with meshed microsculpture (Fig. 49); antenna with articles V-X subquadrate to slightly transverse (Fig. 49); head about as wide as pronotum (Fig. 49); pronotum transverse, slightly narrower than elytra at base, with pubescence directed obliquely laterad from median line of disc and in basal part of median line directed anteriad and laterad, base of disc with small oval impression (Fig. 49); elytra at suture about as long as pronotum and slightly wider at base than pronotum (Fig. 49); abdomen subparallel. MALE. Tergite VIII truncate apically (Fig. 52); sternite VIII elongate and notched apically (Fig. 53). Median lobe of aedeagus with large broad bulbus and narrow triangular tubus in dorsal view, bulbus strongly sinuate laterally (Fig. 51), in lateral view tubus straight and slightly sinuate basally; crista apicalis of bulbus small (Fig. 50); internal sac structures as illustrated (Figs 50, 51). FEMALE. Tergite VIII truncate apically (Fig. 54); sternite VIII arcuate apically (Fig. 55); spermatheca with pitcher-shaped capsule bearing broad and deep apical invagination, stem broad, and coiled (Fig. 56).

##### Distribution.

This species is known only from SK.

##### Natural history.

Adults of this species were collected by sifting wrack on lakeshore beach, and were found in wind-drift on a lake.

#### Dochmonota simulans

Taxon classificationAnimaliaColeopteraStaphylinidae

Klimaszewski & Larson sp. n.

http://zoobank.org/01385C86-C902-4A1D-91BF-5973E19D18F9

Figs 57–64

##### Holotype (male).

Canada, Saskatchewan, Royal Edward Rd., 25 km NW Maple Creek, 5-VI-2011, D. Larson (LFC) 1 male. **PARATYPE** (female): Canada, Saskatchewan, Hwy 21, 17 km N Maple Ceek, 26-VI-2010, saline slough, D. Larson (LFC).

##### Etymology.

The species name is derived from Latin adjective *simulans*-, meaning imitating, in reference to its similarity to the closely related *Dochmonota websteri*.

##### Diagnosis.

Body narrowly subparallel (Fig. 57), length 3.0 mm, uniformly black; head, pronotum and elytra finely and densely punctate, punctures small; pubescence dense; integument moderately glossy, more so on abdomen, with meshed microsculpture; antenna with articles V-VII subquadrate to slightly transverse (Fig. 57); head distinctly narrower than pronotum (Fig. 57); pronotum transverse, distinctly narrower than elytra at base, with pubescence directed obliquely laterad from median line of disc and pubescence in basal part of median line directed anteriad and laterad, base of disc without impression (Fig. 57); elytra at suture distinctly longer than pronotum and wider than pronotum (Fig. 57); abdomen subparallel. MALE. Tergite VIII truncate apically with two small lateral teeth (Fig. 60); sternite VIII elongate and rounded apically (Fig. 61). Median lobe of aedeagus with large suboval bulbus and small triangular tubus in dorsal view, lateral sides of bulbus slightly sinuate (Fig. 59), tubus sinuate in lateral view, crista apicalis of bulbus small (Fig. 58); internal sac structures as illustrated (Figs 58, 59). FEMALE. Tergite VIII truncate apically (Fig. 62); sternite VIII emarginated apically (Fig. 63); spermatheca with subspherical capsule bearing broad invagination, stem irregularly twisted and with swollen apical part (Fig. 64).

##### Distribution.

Adults are known only from SK.

##### Natural history.

The male of this species was captured in June in unspecified habitat, and one female was taken from saline slough, also in June.

#### Dochmonota websteri

Taxon classificationAnimaliaColeopteraStaphylinidae

Klimaszewski & Larson sp. n.

http://zoobank.org/5FE92AA7-3FBB-4C0B-8C63-55C1FB560506

Figs 65–72

##### Holotype (male).

Canada, Saskatchewan, Bigstick Lake, 16 km E Golden Prairie, 1-IX-2011, D. Larson (LFC). **Paratypes**. Canada, Saskatchewan, Bigstick Lake, 16 km E Golden Prairie, 1-IX-2011, D. Larson (LFC) 1 female; Bear Creek at Crane Lake, NE Piapot, 18-VIII-2011, D. Larson (DLC) 1 female. **NON-TYPE**: Canada, Saskatchewan, Bigstick Lake, N Maple Creek, 4-IX-2012, organic mud/sedges, rushes, etc. near water, D. Larson (DLC) 1 male.

##### Etymology.

The species is named for Dr. Reginald R. Webster, close friend of JK, and extraordinary entomologist who “understands aleocharine beetles” and who changed the beetle map of New Brunswick by endless discovery of new species. In memory of our “grappa discussions” and fruitful collaboration.

##### Diagnosis.

Body moderately narrow, subparallel (Fig. 65), length 3.0-3.4 mm, uniformly black with tarsi reddish-brown; antenna with articles I-IV elongate, and V-X slightly transverse (Fig. 65); head, pronotum and elytra finely and densely punctate, punctures small; pubescence dense; integument moderately glossy, more so on abdomen, with meshed microsculpture; head distinctly narrower than pronotum (Fig. 65); pronotum strongly transverse, distinctly narrower than elytra at base, with pubescence directed obliquely laterad from median line of disc and pubescence in basal part of median line directed posteriad and laterad, base of disc without impression (Fig. 65); elytra at suture as long as or slightly longer than pronotum (Fig. 65); abdomen subparallel. MALE. Tergite VIII truncate apically and without apical teeth (Fig. 68); sternite VIII elongate, tapering posteriorly and rounded at apex (Fig. 69). Median lobe of aedeagus with large suboval bulbus and small broad triangular tubus in dorsal view, lateral sides of bulbus gradually narrowed apically (Fig. 67), in lateral view tubus arcuate basally and straight apically and crista apicalis of bulbus small (Fig. 66); internal sac structures as illustrated (Figs 66, 67). FEMALE. Tergite VIII truncate apically (Fig. 70); sternite VIII gradually narrowed apically and truncate, apical margin slightly emarginate (Fig. 71); spermatheca with spherical capsule bearing scarcely seen apical invagination, stem broad, and coiled (Fig. 72).

##### Distribution.

Adults are known only from SK.

##### Natural history.

Most adults of this species were collected from shorelines of eutrophic lakes in June, August and September, and one male was captured in organic mud/sedges, and rushes near water.

##### Comments.

A male from Bigstick Lake had slightly distorted median lobe of aedeagus and was excluded from the type series.

#### Earota dentata

Taxon classificationAnimaliaColeopteraStaphylinidae

(Bernhauer)

(for details and illustrations, see Klimaszewski et al. 2011)

##### Distribution.

**Table:** 

Origin	Nearctic
Distribution	Canada: AB, BC, MB, NB, NL, NS, ON, QC, **SK**. USA: AK, AL, AZ, CO, IA, IL, NC, NJ, NM, OR, VA, WA
New provincial records	CANADA, **Saskatchewan**: Larson Ranch, Hwy 21, 16 km S Maple Creek, 16-VI-2011, D. Larson (DLC) 2 females.
References	Gusarov 2002, Klimaszewski and Winchester 2002, Klimaszewski et al. 2005, 2007b, 2008a, Webster et al. 2009, Majka and Klimaszewski 2008, 2010, Klimaszewski et al. 2011, Bousquet et al. 2013

##### Natural history.

The SK females were captured in June from unspecified habitat. In NL, adults were captured from June to September in the litter of a riparian forest and along the shore of a pond (Klimaszewski et al. 2011). Elsewhere, adults were captured in leaf litter near the margin of a brook in a red maple swamp, in mixed forests of different ages, in river debris, gopher burrows, and under decaying seaweed on a seashore (Klimaszewski and Winchester 2002, Klimaszewski et al. 2005, Majka and Klimaszewski 2008, Webster et al. 2009). Adult activity occurs from April to September.

#### Mocyta breviuscula

Taxon classificationAnimaliaColeopteraStaphylinidae

(Mäklin)

(for details and illustrations, see Klimaszewski et al. 2015a, b)

##### Distribution.

**Table:** 

Origin	Nearctic
Distribution	Canada: AB, BC, MB, NB, NF, NL, NS, NT, **SK**, YT. USA: AK, OR
New provincial records	CANADA, **Saskatchewan**: Larson Ranch, 16 km S Maple Creek: 8-IV-2010, sifting aspen choke-cherry leaf litter, D. Larson (DLC) 2 females; 18-VI-2010, D. Larson (DLC, LFC) 3 males, 2 females; 27-IV-2013, sifting willow, aspen, hawthorn litter near creek, D. Larson (DLC, LFC) 2 males, 6 females; 3-V-2013, aspen litter, D. Larson (DLC, LFC) 2 males, 2 females; 6-V-2013, sifting willow litter, D. Larson (DLC) 2 females; 10-V-2014, under fresh-cut aspen log rings, D. Larson (DLC) 2 males; 13-V-2014, under fresh-cut aspen log rings, D. Larson (DLC) 1 female; 14-V-2014, under fresh-cut aspen log rings, D. Larson (DLC) 3 females; 20-V-2008, D. Larson (DLC) 1 female; 5-6-VI-2013, maple litter, D. Larson (DLC) 3 females; 8-VI-2014, under fresh-cut aspen log rings, D. Larson (DLC) 1 male; 21-VI-2012, decaying bracket fungus on aspen, D. Larson (DLC) 3 females; 10-VIII-2012, aspen/maple litter, D. Larson (DLC) 2 males, 1 female; 8-IX-2012, compost, D. Larson (DLC) 3 females; 28-IX-2010, D. Larson (DLC) 1 male, 3 females; 20-X-2014, sifting willow leaf litter, D. Larson (DLC) 2 males, 7 females; Belanger Creek, Frenchman Valley, 18-X-2014, D. Larson (DLC) 5 males, 4 females; Cypress Hills Park, Center Block: Sucker Creek, 15-V-2013, sifting aspen litter, D. Larson (DLC) 1 male; 16-VI-2011, sifting wrack, D. Larson (DLC) 1 male; 18-VI-2012, sifting aspen litter, D. Larson (DLC) 3 females; Sucker Creek, 23-VI-2014, aspen woodland bracket/gilled fungi, D. Larson (DLC) 1 female; Saskatoon, 27-VII-1972, D. Larson (DLC) 1 female; 7-IX-2014, spruce-aspen, D. Larson (DLC) 1 female; Saskatoon, 7-X-1976, D. Larson (DLC) 1 female.
References	Lohse et al. 1990, Klimaszewski et al. 2005, 2007b, 2008a, b, 2015a, b, Bousquet et al. 2013

##### Natural history.

The SK specimens were captured by sifting aspen litter, maple litter, aspen choke-cherry leaf litter, willow and aspen litter, hawthorn litter near creek, willow leaf litter, under fresh-cut aspen log rings, from decaying woodland bracket/gilled fungi, and from compost, in May through October. In Newfoundland, adults were frequently caught in pitfall traps in various forest types (birch, spruce-lichen, spruce-poplar, fir), in vegetation on coastal sand dunes, on shrubby limestone barrens and in disturbed fields amongst grass and weeds (Klimaszewski et al. 2011). The activity period is June to September. Adults were captured with pitfall traps from June to August in moss and leaf litter in red spruce forest in New Brusnwick and yellow birch/balsam fir forests in southern Quebec (Klimaszewski et al. 2005b, 2007b, 2015b).

#### Mocyta sphagnorum

Taxon classificationAnimaliaColeopteraStaphylinidae

Klimaszewski & Webster

(for details and illustrations, see Klimaszewski et al. 2015b)

##### Distribution.

**Table:** 

Origin	Nearctic
Distribution	Canada: NB, NF, ON, QC, **SK**.
New provincial records	CANADA, **Saskatchewan**: Larson Ranch, Hwy 21, 16 km S Maple Creek: 27-IV-2013, sifting willow-aspen, hawthorn litter near creek, D. Larson (DLC) 1 male, 1 female; 20-V-2008, D. Larson (DLC) 1 female; 25-V-2013, D. Larson (DLC) 1 female; 12-VII-2012, wet grass and weed clippings, D. Larson (DLC) 2 females; 16-VIII-2012, new brome/alfalfa hay, D. Larson (DLC) 1 female; 8-IX-2012, compost, D. Larson (DLC) 1 female; Gull Lake, N town of Gull Lake, 17-V-2014, D. Larson (DLC) 1 female; Cypress Hills Park, Center Block: Highland Trail, 20-V-2013, moist spruce litter near stream, D. Larson (DLC) 2 females; 7-IX-2014, spruce-aspen, D. Larson (DLC) 1 male; 13-IX-2012, sifting spruce litter, D. Larson (DLC) 1 female; Loch Lomond, 21-IX-2011, spruce-aspen litter, D. Larson (DLC) 1 female; Lodgepole Trail, 24-IX-2014, decaying mushrooms, D. Larson (DLC) 1 female.
References	Klimaszewski et al. 2015b

##### Natural history.

In SK, specimens were captured from May through September from willow-aspen litter, hawthorn litter near creek, wet grass and weed clippings, moist spruce litter near stream, spruce litter, spruce-aspen litter, and in decaying mushrooms. In NB, adults were found in sphagnum moss and litter in calcareous eastern white cedar fens and in a black spruce forest (Klimaszewski et al. 2015b). One individual was collected from mouldy conifer duff at the base of a large pine in a mixed forest (Klimaszewski et al. 2015b). Adults were found in April and May in New Brunswick, and June to August elsewhere. This species often seems to be associated with moist sphagnum moss (Klimaszewski et al. 2015b).

##### Comments.

Males of this species can be mixed up with those of *Mocyta breviuscula* and positive identification may only be possible with female association as *Mocyta* are definitively identified by the shape of the spermatheca.

#### Nehemitropia lividipennis

Taxon classificationAnimaliaColeopteraStaphylinidae

(Mannerheim)

(for details and illustrations, see Klimaszewski et al. 2007a, 2011)

##### Distribution.

**Table:** 

Origin	Palaearctic, adventive in Canada
Distribution	Canada: NB, NL, NS, ON, PE, QC, **SK**. USA: CA, LA, MA, MN, NE, NM, NY, PA, VT, TX
New provincial records	CANADA, **Saskatchewan**: Saskatoon, 26-IX-1976, D. Larson (DLC) 1 male, 1 female.
References	Moore and Legner 1975, Klimaszewski et al. 2007a, Majka and Klimaszewski 2010, Klimaszewski et al. 2011, Bousquet et al. 2013

##### Natural history.

The SK specimens were captured from an unspecified habitat in September. In NL, one specimen was collected in October from an unspecified habitat (Klimaszewski et al. 2011). Elsewhere in North America, adults were captured in open fields and pastures, in organic debris including dead grass, in caribou, horse and cow dung, in open marsh, maple/beech forest, the edge of an oak forest, and in the nest of *Microtus pennsylvanicus* (Ord) (Klimaszewski et al. 2007a, 2011).

#### Philhygra falcifera

Taxon classificationAnimaliaColeopteraStaphylinidae

Lohse

(for details and illustrations, see Lohse et al. 1990)

##### Distribution.

**Table:** 

Origin	Nearctic
Distribution	Canada: MB, **SK**
New provincial records	CANADA, **Saskatchewan**: Cypress Hills Park, Highland Trail, 10-VI-2013, treading quaking moss, *Typha*, *Equisetum*, D. Larson (DLC) 1 male.
References	Lohse et al. 1990, Bousquet et al. 2013

##### Natural history.

The SK male was captured in June by treading quaking moss, *Typha*, and *Equisetum*. The MB specimens were captured in June and August, from unspecified habitat (Lohse et al. 1990).

#### Philhygra subpolaris

Taxon classificationAnimaliaColeopteraStaphylinidae

(Fenyes)

(for diagnosis and illustrations, see Fenyes 1909, Klimaszewski et al. 2016)

##### Distribution.

**Table:** 

Origin	Nearctic
Distribution	Canada: AB, **SK**. USA: AZ
New provincial records	CANADA, **Saskatchewan**: Larson Ranch, Hwy 21, 16 km S Maple Creek, 9-V-2013, sifting willow/grass litter, D. Larson (DLC) 1 male; Cypress Lake Park, 16-VI-2011, sifting wrack, D. Larson (DLC) 1 female.
Reference	Fenyes 1909, Klimaszewski et al. 2016

##### Natural history.

In SK, one male was captured in May by sifting willow/grass litter, and one female was sifted from wrack on a lakeshore in June. In AB, adults were caught in window traps attached to aspen snags in a boreal aspen stand harvested 2 years previously, and in pitfall traps deployed in canola fields. Adults were collected in July (Klimaszewski et al. 2016a).

#### Schistoglossa blatchlyei

Taxon classificationAnimaliaColeopteraStaphylinidae

(Bernhauer & Scheerpeltz)

(for diagnosis and illustrations, see Klimaszewski et al. 2009a)

##### Distribution.

**Table:** 

Origin	Nearctic
Distribution	Canada: MB, NB, NT, ON, QC, **SK**, YT; USA: AK, IN
New provincial records	CANADA, **Saskatchewan**: Cypress Hills Park, Center Block, Highland Trail, 10-VI-2013, treading quaking moss, *Typha* and *Equisetum* in June, D. Larson (DLC) 1 male.
Reference	Blatchley 1910, Bernhauer and Scheerpeltz 1926, Klimaszewski et al. 2009a, Bousquet et al. 2013

##### Natural history.

In SK, one male was captured in June by treading quaking moss, *Typha* and *Equisetum*.

#### Strigota ambigua

Taxon classificationAnimaliaColeopteraStaphylinidae

(Erichson)

(for diagnosis and illustrations, see Klimaszewski et al. 2011)

##### Distribution.

**Table:** 

Origin	Nearctic
Distribution	Canada: LB, NF, NS, ON, PE, QC, **SK**, YT. USA: CA, CO, CT, IA, KS, MO, NC, NJ, NM, NY, TX
New provincial records	CANADA, **Saskatchewan**: Great Sand Hills, 50.9°N, 109.11°W, Bowie Ranch, 8-VII-2013, Larson (DLC) 1 female; Larson Ranch, 16 km S Maple Creek, 9-VII-2014, D. Larson (DLC) 1 female; 12 km NE Gull Lake, *Scirpus* wrack, saline pond, 25-V-2011, D. Larson (DLC) 1 male; Tompkins, Sidewood Rad, 15-IX-2014, D. Larson (DLC) 1 male.
References	Gusarov 2003, Majka et al. 2008, Majka and Klimaszewski 2010, Brunke et al. 2012, Bousquet et al. 2013, Webster et al. 2016b

##### Natural history.

In SK, one specimen was found in *Scirpus* wrack on the shore of saline pond, and three others were found in unspecified habitats in May, July and September. In NB, one specimen was found under a cobblestone on moist sand on a lake margin (Webster et al. 2016b). This widespread species occurs in open habitats, including dunes, beaches, limestone barrens, soybean fields, old fields, open gaps in spruce forest, riverbanks and groundhog burrows (Brunke et al. 2012).

#### Strigota obscurata

Taxon classificationAnimaliaColeopteraStaphylinidae

Klimaszewski & Brunke

(for diagnosis and illustrations, see Brunke et al. 2012)

##### Distribution.

**Table:** 

Origin	Nearctic
Distribution	Canada: NB, ON, **SK**
New provincial records	CANADA, **Saskatchewan**: Cypress Lake, E dam, wind-drift, 9-V-2012, D. Larson (DLC) 1 female.
References	Brunke et al. 2012, Bousquet et al. 2013, Webster et al. 2016b

##### Natural history.

In SK, one female was captured in May from wind-drift on the lake. In NB, *Strigota obscurata* was found in flood debris on a river margin, on soil at the base of grass in a residential lawn, and captured in a Lindgren funnel trap in an old jack pine forest (Webster et al. 2016b). Brunke et al. (2012) reported this as the most common species in southern Ontario soybean fields, often occurring in open habitats with *Strigota ambigua*.

### Tribe AUTALIINI Thomson

#### Autalia rivularis

Taxon classificationAnimaliaColeopteraStaphylinidae

(Gravenhorst)

(for diagnosis and illustrations, see Klimaszewski et al. 2011)

##### Distribution.

**Table:** 

Origin	Palaearctic, adventive in Canada
Distribution	Canada: AB, BC, LB, NB, NF, NS, ON, QC, **SK.** USA: CA, MI, MN, NH, NY, OR
New provincial records	CANADA, **Saskatchewan**: Cypress Hills Park, C Block, Sucker Creek, 23-VIII-2012, moose dung, D. Larson (DLC) 2 females; Larson Ranch, Hwy 21, 16 km S Maple Creek: 21-VI-2012, under bark of dead aspen, D. Larson (DLC) 1 female; 1-IX-2012, compost, D. Larson (DLC) 1 male.
References	Hoebeke 1988, Klimaszewski et al. 2005, Majka and Klimaszewski 2010, Klimaszewski et al. 2011, Bousquet et al. 2013

##### Natural history.

The SK specimens were found in moose dung, under bark of dead aspen, and in compost in June, August and September. In NL, adults were collected in July using flight intercept traps in mixedwood forest and carrion traps on coastal shrubby barrens (Klimaszewski et al. 2011). Elsewhere, adults were collected in July and August from red spruce dominated regenerating forest in NB (Klimaszewski et al. 2005). In Europe, this species is very common in cow dung and rotting organic debris.

### Tribe FALAGRINI Mulsant & Rey

#### Falagria caesa

Taxon classificationAnimaliaColeopteraStaphylinidae

Erichson

(for diagnosis and illustrations, see Klimaszewski et al. 2013b, Hoebeke 1985 [as Falagria sulcata (Paykull)])

##### Distribution.

**Table:** 

Origin	Palaearctic, adventive in Canada
Distribution	Canada: AB, BC, NB, ON, QC, **SK**. USA: IL, MA, MD, NJ, NY, UT, VA
New provincial records	CANADA, **Saskatchewan**: Larson Ranch, Hwy 21, 16 km S Maple Creek: 1-IX-2012, compost, D. Larson (DLC, LFC) 1 female, 1 sex undetermined; 22-27-VI-2005, D. Larson (DLC) 1 sex undetermined; 17-IX-2012, compost, D. Larson (LFC) 1 male; Cypress Hills Lake: E dam, wind-drift, 9-V-2012, D. Larson (DLC) 1 sex undetermined; E end, sifting wrack, 31-VII-2012, D. Larson (DLC, LFC) 1 female, 5 sex undetermined; Crane Lake, NE Piapot., beach wrack, 28-VIII-2011, D. Larson (DLC, LFC) 1 male, 3 sex undetermined; Bigstick Lake, 16 km E Golden Prairie, 21-IX-2011, D. Larson (DLC) 1 sex undetermined; Saskatoon, 26-IX-1976, compost, D. Larson (DLC) 1 sex undetermined.
References	Hoebeke 1985, Klimaszewski et al. 2010, Webster et al. 2012, Klimaszewski et al. 2013b, Bousquet et al. 2013

##### Natural history.

The SK specimens were found in compost, wind drift, and beach wrack, from June through September. In North America, this species is associated with decaying plant material such as compost, mouldy corncobs, cornhusks, weeds, haystacks and rotting fungi (Hoebeke 1985, Webster et al. 2012, Klimaszewski et al. 2013b).

##### Comments.

This species is well established in northeastern and western North America (Hoebeke 1985). It was listed in North America as *Falagria sulcata* (Hoebeke 1985, Campbell and Davies 1991, Klimaszewski et al. 2010, Webster et al. 2012). The oldest record of this adventive species in SK is that of 1976.

#### Myrmecocephalus arizonicus

Taxon classificationAnimaliaColeopteraStaphylinidae

(Casey)

(for diagnosis and illustrations, see Hoebeke 1985)

##### Distribution.

**Table:** 

Origin	Nearctic
Distribution	Canada: AB, BC, **SK**. USA: AZ, CO, ID, NM, UT
New provincial records	CANADA, **Saskatchewan**: Larson Ranch, Hwy 21, 16 km S Maple Creek: 22-V-2008, D. Larson (DLC) 1 sex undetermined; 5-6-VI-2013, D. Larson (DLC) 1 sex undetermined; 15-30-VI-2006, D. Larson (DLC) 1 male; 18-VI-2001, D. Larson (LFC) 1 male; Cypress Hills, Center Block: Hidden Valley, 1-VI-1999, D. Larson (DLC) 1 male, 1 sex undetermined; 4-VI-2006, pine clearcut, D. Larson (DLC, LFC) 1 male, 1 female; Ski Lodge, 25-VI-2004, recently dead white spruce, D. Larson (DLC, LFC) 1 female, 2 sex undetermined; fire guard, 29-IX-2013, sifting moss and pine litter, D. Larson (DLC) 1 sex undetermined.
References	Hoebeke 1985, Bousquet et al. 2013

##### Natural history.

The SK specimens were found in pine clearcut, on recently dead white spruce, and in moss and pine litter in May, June and September. Elsewhere, specimens were collected from under bark of logs, from leaf litter, flood debris and wet moss, from soil along a stream, from fungus (*Fomitopsis pinicola*, *Fomes robineae*), and from a squirrel midden (Hoebeke 1985).

### HOMALOTINI Heer

#### Agaricochara pulchra

Taxon classificationAnimaliaColeopteraStaphylinidae

Klimaszewski & Larson sp. n.

http://zoobank.org/9BD29B8C-4286-4D39-A0AB-0B4DC688AE8E

Figs 73–79

##### Holotype (male).

Canada, Saskatchewan, Larson Ranch, Hwy 21, 16 km S Maple Creek, 12-IX-2013, mouldy aspen log, D. Larson (LFC). **Paratypes**. Canada, Saskatchewan, Larson Ranch, Hwy 21, 16 km S Maple Creek, 12-IX-2013, mouldy aspen log, D. Larson (DLC, LFC) 1 male, 2 females, 11 sex undetermined; Cypress Hills Pk., Center Block, Hidden Valley, 1-VI-1999, D. Larson (DLC) 1 female; Cypress Hills Pk., Center Block, Sucker Cr., 18-VII-2012, sifting aspen litter, D. Larson (DLC) 1 female.

##### Etymology.

A Latin feminine adjective *pulchra*, meaning beautiful, in reference to the body shape and beautiful colour of this species.

##### Diagnosis.

**Figures 73–79.**
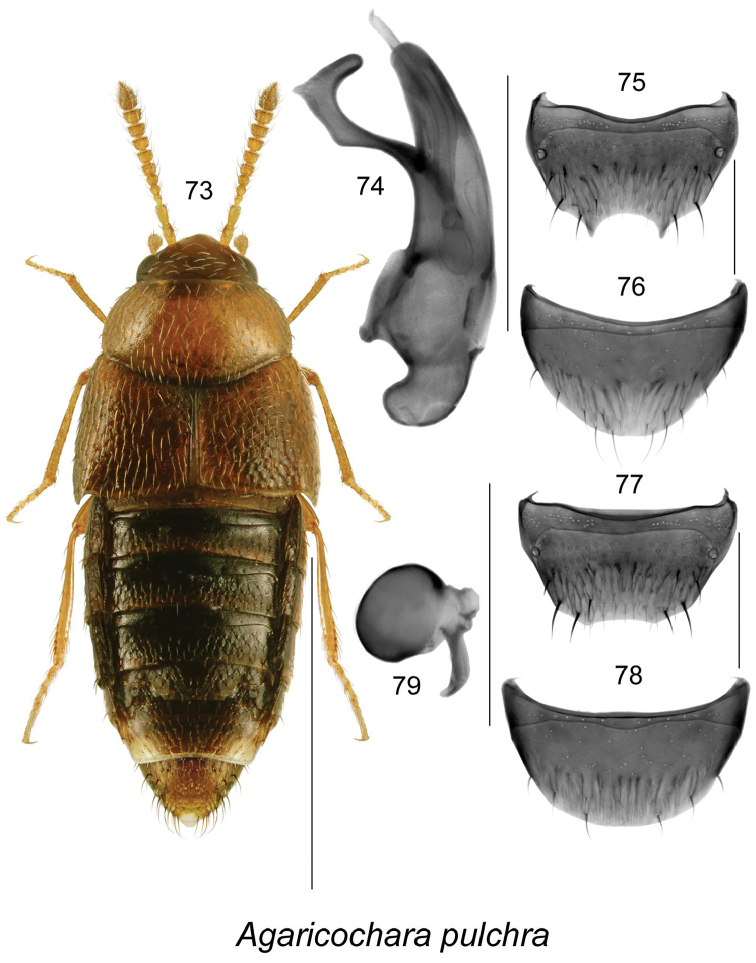
*Agaricochara pulchra* Klimaszewski & Larson, sp. n.: **73** habitus in dorsal view **74** median lobe of aedeagus in lateral view **75** male tergite VIII **76** male sternite VIII **77** female tergite VIII **78** female sternite VIII **79** spermatheca. Scale bar for habitus = 1 mm, and the remaining scale bars = 0.2 mm.

Body minute, narrowly oval, moderately convex, length 1.4-1.6 mm (Fig. 73); head and abdomen (except for apex) piceous, pronotum and elytra reddish-yellow, elytra with darker scuteller and posterior angle sections, legs and antennae except for the last article yellow (Fig. 73); punctation on forebody fine and sparse, those on elytra asperate; pubescence on pronotum directed posteriad (Fig. 73); abdomen tapering apically with scale-like sculpture (Fig. 73); antennae gradually broadening apically, articles V-X transverse (Fig. 73). MALE. Tergite VIII emarginate medially and with two lateral teeth (Fig. 75); sternite VIII rounded apically (Fig. 76); median lobe of aedeagus with subapical process angular subapically (Fig. 74). FEMALE. Tergite and sternite VIII shallowly concave apically (Fig. 77); sternite VIII transverse and broadly arcuate apically (Fig. 78); spermatheca small, capsule spherical (Fig. 79).

##### Distribution.

Known only from SK. This constitutes new genus record for Canadian fauna.

##### Natural history.

Adults were collected from mouldy aspen logs in September and by sifting aspen litter in July.

##### Comments.

Seevers (1951) considered *Agaricochara* Kraatz

 as a subgenus of *Gyrophaena* Mannerheim, but Ashe (1984) elevated it to the generic rank. We have followed Ashe (1984) in treating this taxon as a genus. There are two species of *Agaricochara* in Europe and six in North America (Seevers 1951). No member of either group of species matches our new species from SK, which has very distinctively shaped tubus of the median lobe of the aedeagus with ventral process-like projection angularly bent subapically and directed dorsally (Fig. 74).

#### Gyrophaena lobata

Taxon classificationAnimaliaColeopteraStaphylinidae

Casey

(for diagnosis and illustrations, see Seevers 1951, Klimaszewski et al. 2009b)

##### Distribution.

**Table:** 

Origin	Nearctic
Distribution	Canada: NB, **SK**. USA: DC, IL, IN, KA, MI, NY, WA, WI
New provincial records	CANADA, **Saskatchewan**: Larson Ranch, Hwy 21, 16 km S Maple Creek, 29-VIII-2014, D. Larson (DLC) 1 male.
References	Casey 1906, Seevers 1951, Klimaszewski et al. 2009b, Bousquet et al. 2013

##### Natural history.

The SK specimen was collected in August from unspecified habitat. In NB, adults were captured in gilled mushrooms in mixed and hardwood forests from July through September by sifting mushrooms and aspirating specimens (Klimaszewski et al. 2009b).

#### Gyrophaena subnitens

Taxon classificationAnimaliaColeopteraStaphylinidae

Casey

(for diagnosis and illustrations, see Seevers 1951, Klimaszewski et al. 2009b)

##### Distribution.

**Table:** 

Origin	Nearctic
Distribution	Canada: MB, ON, **SK**. USA: IL, KS, ME, MN, MO, NY, WI
New provincial records	CANADA, **Saskatchewan**: Cypress Hills Park, Block Fire, Sucker Creek, 23-VI-2014, aspen woodland bracket/gilled fungi, D. Larson (LFC) 1 male; Maple Creek, Hwy 21, 16 km S, 18-VII-2003, D. Larson (DLC) 1 male, 1 female.
References	Casey 1906, Seevers 1951, Klimaszewski et al. 2009b, Bousquet et al. 2013

##### Natural history.

Two SK specimens were found in aspen woodland on bracket/gilled fungi, in June and July. In NB, specimens were collected by sifting in June from sun-exposed gilled mushrooms on stump in 8.5-year-old regenerating mixed forest and red oak (Klimaszewski et al. 2009b).

#### Leptusa gatineauensis

Taxon classificationAnimaliaColeopteraStaphylinidae

Klimaszewski & Pelletier

(for diagnosis and illustrations, see Klimaszewski et al. 2004)

##### Distribution.

**Table:** 

Origin	Nearctic
Distribution	Canada: AB, BC, NB, NF, NS, ON, QC, **SK**
New provincial records	CANADA, **Saskatchewan**: Cypress Hills Park, Center Block, 1-VI-2004, Hooper & Larson (DLC) 1 male, 1 sex undetermined; Larson Ranch, Hwy 21, 16 km S Maple Creek: 27-IV-2013, sifting willow, aspen, hawthorn near creek, D. Larson (DLC, LFC) 1 male, 6 sex undetermined; 14-V-2014, under bark/in polypore fungus on aspen, D. Larson (DLC) 3 sex undetermined; 5-6-VI-2013, maple litter, D. Larson (DLC) 2 sex undetermined; 6-VI-2013, D. Larson (DLC) 1 sex undetermined; 8-VI-2007, under bark/in polypore fungus on aspen, D. Larson (DLC) 1 female, 1 sex undetermined; 21-VI-2012, under bark of dead aspen, D. Larson (DLC) 1 sex undetermined.
References	Klimaszewski et al. 2004, McLean et al. 2009a, b, Bousquet et al. 2013

##### Natural history.

The SK specimens were collected from willow, aspen, and hawthorn litter near creek, under bark of dead aspen, in polypore fungus on aspen, in May and June. Elsewhere, two specimens were captured in May on *Polyporus betulinus*, one by general sweeping in deciduous forest, and one in June in red spruce/hemlock mature forest (Klimaszewski et al. 2004). A few specimens were collected by funnel trap in Stanley Park, Vancouver (McLean et al. 2009a, b).

### Tribe HYPOCYPHTINI Laporte

#### Cypha crotchi

Taxon classificationAnimaliaColeopteraStaphylinidae

(Horn)

(for illustrations, see Klimaszewski et al. 2008b)

##### Distribution.

**Table:** 

Origin	Nearctic
Distribution	Canada: AB, BC, **SK**
New provincial records	CANADA, **Saskatchewan**: Cypress Hills Park, Center Block, Lodgepole Trail, 24-IX-2014, decaying mushrooms, D. Larson (DLC) 1 male.
References	Klimaszewski et al. 2008b, Bousquet et al. 2013

##### Natural history.

The SK male was found in September in decaying mushrooms.

#### Cypha inexpectata

Taxon classificationAnimaliaColeopteraStaphylinidae

Klimaszewski & Godin

(for illustrations, see Klimaszewski et al. 2008b)

##### Distribution.

**Table:** 

Origin	Nearctic
Distribution	Canada: ON, **SK**, YT
New provincial records	CANADA, **Saskatchewan**: Cypress Hills Park, Center Block: Lodgepole Trail, 24-IX-D. Larson (DLC) 1 female; Belanger Creek, 14-X-2014, mossy hummocks bordering marsh and spruce forest, D. Larson (DLC, LFC) 4 females; Belanger Creek, Frenchman Valley, 18-X-2014, mossy hummocks near creek, D. Larson (LFC) 1 male.
References	Klimaszewski et al. 2008b, Bousquet et al. 2013

##### Natural history.

In SK, specimens were collected from mossy hummocks at the border between a marsh and spruce forest, and mossy hummocks near creek, in September and October.

#### Oligota inflata

Taxon classificationAnimaliaColeopteraStaphylinidae

(Mannerheim)

Figs 80–86

##### Diagnosis.

**Figures 80–86.**
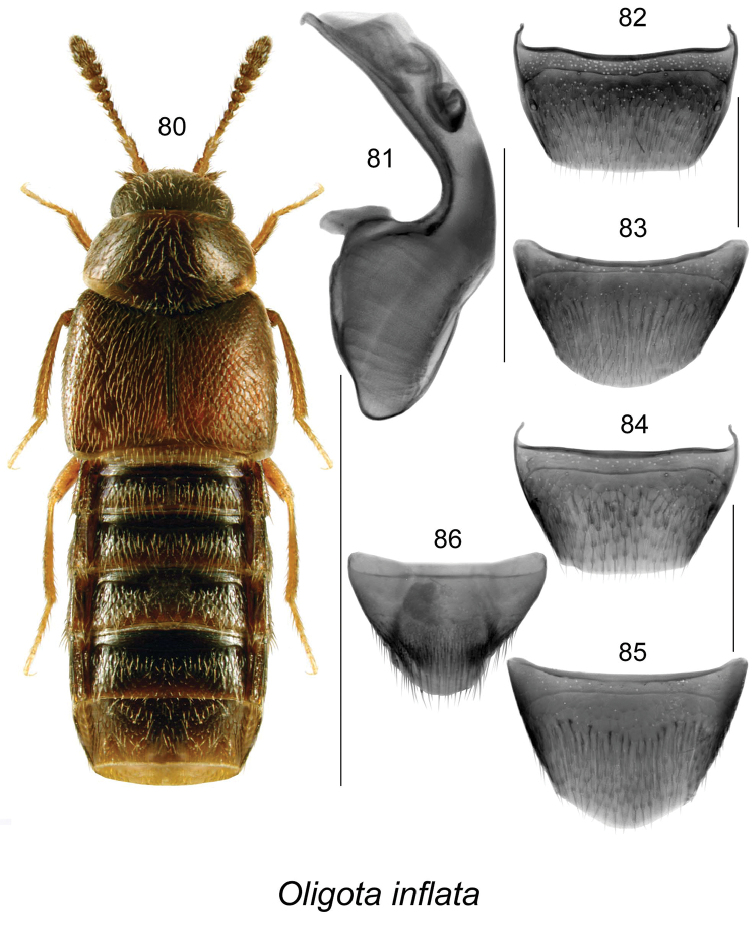
*Oligota inflata* (Mannerheim): **80** habitus in dorsal view **81** median lobe of aedeagus in lateral view **82** male tergite VIII **83** male sternite VIII **84** female tergite VIII **85** female sternite VIII **86** female pygidium. Scale bar for habitus = 1 mm, and the remaining scale bars = 0.2 mm.

Body length 1.4–1.5 mm, compact, subparallel, piceous to nearly black, with legs/tarsi, three basal antennal articles, maxillary palps, posterior edge of elytra, and tip of abdomen yellowish brown (Fig. 80); moderately glossy; integument with microsculpture mesh-like on head and pronotum, and coarse, scale-like on elytra and abdomen (Fig. 80); pubescence sparse and long; head transverse with pubescence directed anteriad; eyes large, and protruding (Fig. 80); antennae with four apical articles forming loose club, articles VI–VII narrow and VIII-X moderately to strongly transverse (Fig. 80); pronotum strongly transverse, lateral margins strongly converging apicad, pubescence directed obliquely laterad (Fig. 80); elytral margins broadly arcuate laterally with pubescence directed obliquely laterad (Fig. 80); abdomen gradually narrowed apically. MALE. Tergite VIII truncate apically (Fig. 82); sternite VIII with apical margin arcuate (Fig. 83); median lobe of aedeagus with tubus long, arcuate, and apex hooked ventrally in lateral view, bulbus moderately long with small and irregularly oval crista apicalis (Fig. 81). FEMALE. Tergite VIII truncate apically (Fig. 84); sternite VIII broadly rounded and slightly produced apically (Fig. 85); pygidium as illustrated (Fig. 86); spermatheca not found.

##### Distribution.

**Table:** 

Origin	Palaearctic, adventive in Canada
Distribution	Canada: **SK**
New North American, Canadian and provincial records	CANADA: **Saskatchewan**, Larson Ranch, Hwy 21, 16 km S Maple Creek: 14-V-2013 (DLC) 1 female; 22-27-VI-2005 (DLC) 1 female; 16-VIII-2012, new brome/alfalfa hay, D. Larson (DLC, LFC) 2 males, 5 females; 1-IX-2012, compost, D. Larson (DLC, LFC) 5 males, 9 females.
References	Mannerheim 1830, Williams 1978

##### Natural history.

The SK specimens were found in compost and new brome/alfalfa hay. Collecting period: June, August and September

##### Comments.

*Oligota inflata* is a Palaearctic species known from Europe, N. Africa, Congo, Egypt, and Brazil. It is reported here for the first time from North America.

### Tribe LOMECHUSINI Fleming

#### Zyras obliquus

Taxon classificationAnimaliaColeopteraStaphylinidae

(Casey)

(for illustrations, see Klimaszewski et al. 2011)

##### Distribution.

**Table:** 

Origin	Nearctic
Distribution	Canada: AB, BC, MB, NB, NF, NS, ON, QC, **SK**. USA: MI, MO, NH, NY, OR
New provincial records	CANADA, **Saskatchewan**: Larson Ranch, 16 km S Maple Creek, 1-15-VI-2005, D. Larson (DLC) 1 sex undetermined; Cypress Hills Park, Center Block, 13-VI-2003, D. Larson (DLC, LFC) 2 sex undetermined
References	Casey 1893, Klimaszewski et al. 2005, Webster et al. 2009, Majka and Klimaszewski 2010, Klimaszewski et al. 2011, Bousquet et al. 2013

##### Natural history.

The SK specimens were collected in June from unspecified habitat.

### Tribe OXYPODINI C.G. Thomson

#### Ganthusa eva

Taxon classificationAnimaliaColeopteraStaphylinidae

Fenyes

(for illustrations, see Klimaszewski et al. 2014)

##### Distribution.

**Table:** 

Origin	Nearctic
Distribution	Canada: AB, BC, **SK**, YT. USA: CA
New provincial records	CANADA, **Saskatchewan**: Cypress Hills Park, Center Block, Sucker Creek, 20-V-2013, lodgepole pine litter, D. Larson (DLC) 1 male
References	Fenyes 1909, Klimaszewski and Winchester 2002, Majka and Klimaszewski 2008, Bousquet et al. 2013, Klimaszewski et al. 2014

##### Natural history.

In SK, one specimen was collected in May from lodgepole pine litter. Elsewhere, adults were captured in clear-cut Sitka spruce forest on Vancouver Island and in moss and gravel at the edge of small pools at other localities in the interior of British Columbia (Klimaszewski and Winchester 2002). Additional specimens were found in British Columbia in a 1-year-old harvested Douglas-fir stand. In west-central Alberta, adults were collected in pitfall traps deployed in Upper Cordilleran coniferous forests, including subxeric lodgepole pine forests, mesic white spruce and lodgepole pine stands and spruce-dominated subhygric and hygric forests, but not in deciduous-dominated forest or in grassy or shrubby meadows (Klimaszewski et al. 2014). In Alberta, adults also emerged from lodgepole pine trees infested by bark beetles (Klimaszewski et al. 2014). In the Yukon Territory, adults were found in a squirrel midden in spring, probably overwintering, and in a coniferous woodchip pile (Klimaszewski et al. 2014).

#### Hylota ochracea

Taxon classificationAnimaliaColeopteraStaphylinidae

Casey

(for illustrations, see Klimaszewski et al. 2006)

##### Distribution.

**Table:** 

Origin	Nearctic
Distribution	Canada: NB, NS, NT, ON, QC, **SK**. USA: NY
New provincial records	CANADA, **Saskatchewan**, Larson Ranch, Hwy 21, 16 km S Maple Creek: 25-VI-2008, carrion trap, D. Larson (DLC) 1 female; 4-VIII-1998, D. Larson (DLC) 1 female; 27-VIII-2012, pigeon coop, D. Larson (DLC) 1 male
References	Casey 1906, Klimaszewski et al. 2006, Majka et al. 2006, Webster et al. 2009, Bousquet et al. 2013, Webster et al. 2016b

##### Natural history.

In SK, one specimen was collected from pigeon coop, one from carrion trap, and one from unspecified habitat. In NB, *Hylota ochracea* was a common inhabitant of barred owl nests (Webster et al. 2009). Barred owl nests were in tree holes (usually in large trees) and in artificial nest boxes (Webster et al. 2009). Adults of *Hylota ochracea* occurred in the nest contents, which usually consisted of rich decaying organic material with bones, fur, owl pellets, portions of dead prey items (mice, squirrels, small birds), and often the contents had a strong urine smell. This species was also found in the nest contents of the great horned owl. Majka et al. (2006) reported this species from the nests of the boreal owl, *Aegolius funereus richardsoni* (Bonaparte) and northern saw-whet owl, *Aegolius acadicus* (Gmelin) in Nova Scotia. Interestingly, *Hylota ochracea* was also common among decaying vegetables inside a plastic compost bin, which in some respects mimics the conditions found within a tree hole occupied by an owl (Webster et al. 2009). Only one adult of *Hylota ochracea* has been captured in New Brunswick in a habitat other than a tree hole or other enclosed situation; in drift material along a river margin (Webster et al. 2009). Adults were collected in May, June, August and September.

#### Oxypoda demissa

Taxon classificationAnimaliaColeopteraStaphylinidae

Casey

(for illustrations, see Klimaszewski et al. 2006, 2011)

##### Distribution.

**Table:** 

Origin	Nearctic
Distribution	Canada: LB, NB, NF, NS, ON, QC, **SK**, YT
New provincial records	CANADA, **Saskatchewan**, Larson Ranch, Hwy 21, 16 km S Maple Creek: Apr., 27-IV-2013, sifting willow, aspen, hawthorn litter near creek, D. Larson (DLC) 1 male, 1 female; 21-VI-2012, under bark of dead aspen, D. Larson (DLC) 1 female; 20-X-2014, sifting willow leaf litter, D. Larson (DLC) 1 female.
References	Casey 1911, Klimaszewski et al. 2006, Webster et al. 2009, Klimaszewski et al. 2011, Bousquet et al. 2013

##### Natural history.

In SK, specimens were captured in willow, aspen, and hawthorn litter near creek, and under bark. In New Brunswick, adults were captured in moist leaf litter on the margin of a vernal pond in a mixed forest, among leaves and sedges on pond margin, in moist grass litter and sphagnum in *Carex* marsh, among sedges along margin of small spring-fed brook in a mature hardwood forest and among leaf litter and grass on hummocks in a wet alder (*Alnus* sp.) swamp (Webster et al. 2009). In Nova Scotia, this species was reported from litter of *Alnus* clumps (Klimaszewski et al. 2006). A number of adults were collected with a net during late afternoon (15:00 to 18:00 h) flights (Webster et al. 2009). Adults were captured from April to July, and in October. Collection method: sifting leaf litter, some collected in flight with net during evening.

#### Oxypoda domestica

Taxon classificationAnimaliaColeopteraStaphylinidae

Klimaszewski & Larson sp. n.

http://zoobank.org/028AB4CE-90D8-4A0F-A833-E5E75466FEFD

Figs 87–91

##### Holotype (male).

Canada, Saskatchewan, Larson Ranch, Hwy 21, 16 km S Maple Creek, 22-IV-2012, D. Larson (LFC). **Paratype**. Canada, Saskatchewan, Larson Ranch, Hwy 21, 16 km S Maple Creek, 1-IV-2012, D. Larson (CNC) 1 male.

##### Etymology.

The name of this species is derived from Latin feminine adjective *domestica*-, meaning domestic, in reference to the capture of the type specimens in the vicinity of the farmstead.

##### Diagnosis.

**Figures 87–91.**
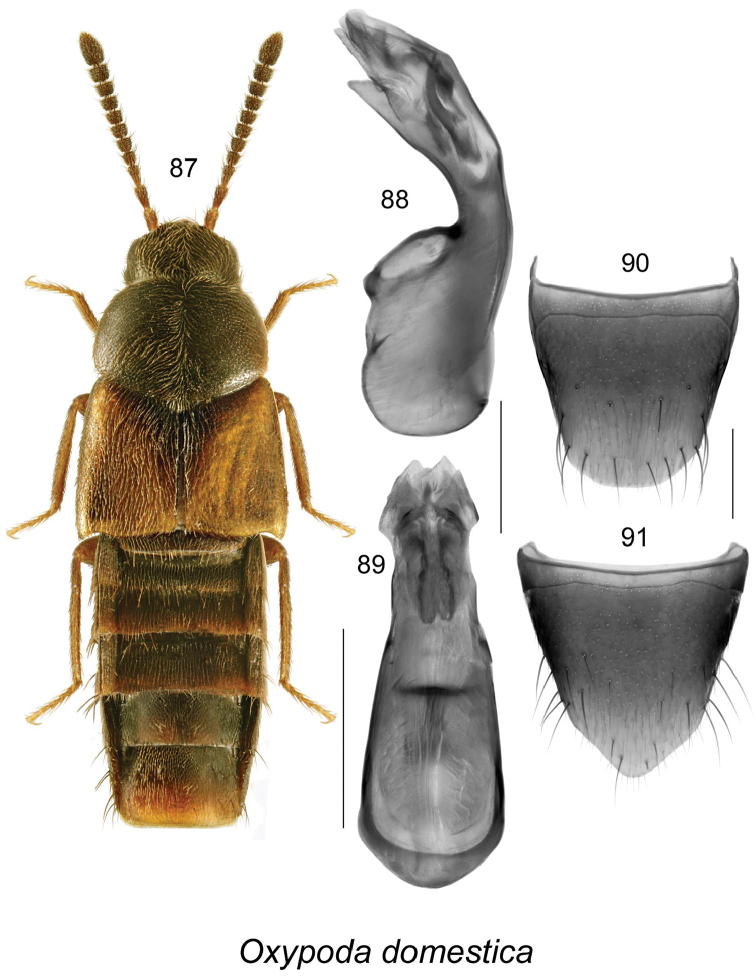
*Oxypoda domestica* Klimaszewski & Larson, sp. n.: **87** habitus in dorsal view **88** median lobe of aedeagus in lateral view, and **89** in dorsal view **90** male tergite VIII **91** male sternite VIII. Scale bar for habitus = 1 mm, and the remaining scale bars = 0.2 mm.

Body length 3.4-3.6 mm, narrowly subparallel, broadest at posterior elytra, abdomen subparallel (Fig. 87); piceous with legs, basal antennal article, and two narrow oblique sections of elytra yellowish-brown (the extent of this section is variable) (Fig. 87); pubescence and punctation of forebody dense; integument with isodiametric microsculpture. Head distinctly broader than half of pronotal width (Fig. 87); eyes large, longer than postocular area in dorsal view; antennae slender, antennomeres I-III strongly elongate, IV slightly elongate, V subquadrate, VI-X moderately transverse (Fig. 87); pronotum moderately convex, strongly transverse and about one fifth broader than long, broadest in basal third, pubescence directed anteriad apically along midline and obliquely posteriad from midline of disc elsewhere (Fig. 87); elytra slightly broader than pronotum and at suture about as long as pronotum, pubescence directed approximately straight posteriad (Fig. 87); abdomen subparallel and slightly tapering apically (Fig. 87). MALE. Tergite VIII transverse and broadly arcuate apically, antecostal suture approximately straight (Fig. 90); sternite VIII triangularly produced apically, antecostal suture slightly sinuate (Fig. 91); median lobe of aedeagus with narrowly oval bulbus and broad and subparallel tubus in dorsal view (Fig. 89); ventral margin of tubus slightly sinuate and with apex triangular in lateral view (Fig. 88); internal sac with elongate subapical structures (Figs 88, 89); bulbus with ovally elongate crista apicalis (Fig. 88). FEMALE. Unknown.

##### Natural history.

The two males were captured in April in an unspecified habitat near a farmstead.

##### Comments.

This species is very similar externaly to *Oxypoda irrasa* Mäklin, from which it may be distinguished by the shape of tubus of median lobe of aedeagus with slightly sinuate ventral margin and triangular apical part in lateral view (Fig. 85). In *Oxypoda irrasa*, tubus of median lobe of aedeagus is angularly bent ventrally and apical part is evenly narrowly elongate. For illustrations of *Oxypoda irrasa*, see Klimaszewski et al. (2006).

#### Oxypoda irrasa

Taxon classificationAnimaliaColeopteraStaphylinidae

Mäklin

(for illustrations, see Klimaszewski et al. 2006)

##### Distribution.

**Table:** 

Origin	Nearctic
Distribution	Canada: AB, **SK**, YT. USA: AK, OR
New provincial records	CANADA, **Saskatchewan**, Larson Ranch, Hwy 21, 16 km S Maple Creek: Apr., 22-IV-2010, dam, D. Larson (DLC) 1 female; 28-IV-2011, on snowbank (DLC) 1 female; 15-VII-2014, decaying polypore mushrooms (DLC) 1 male; 7-X-2010, (LFC) 1 male; Cypress Hills Park, Center Block fire guard, 8-VIII-2013: gilled mushroom, D. Larson (DLC, LFC) 1 male, 5 females; 18-VIII-2014, old polypore fungus on dead lodgepole pine stump (DLC) 2 males, 1 female; Highland Trail, 2-X-2014, gilled mushroom (LFC) 1; 7-X-2014, spruce-aspen (DLC) 1 female; 10-X-2013, decaying mushrooms (DLC) 2 females.
References	Mäklin 1953, Lohse and Smetana 1985, Klimaszewski et al. 2006, 2008a, Bousquet et al. 2013

##### Natural history.

In SK, specimens were captured on decaying and old polypore mushrooms in lodgepole pine and spruce-aspen habitats in March, July, August and September. One specimen was captured on snowbank in March. Elsewhere, adults were captured from May through August with most of the specimens taken in August (Klimaszewski et al. 2006). At the EMEND site (Alberta), adults of *Oxypoda irrasa* (n = 519), like those of *Oxypoda grandipennis*, were found in all cover types and all retention treatments but were most abundant in unharvested stands (Klimaszewski et al. 2006). *Oxypoda irrasa* was collected from May through August at EMEND (Alberta), however a few individuals were collected in May through July (Klimaszewski et al. 2006). This species was most abundant in August. Collecting methods: unbaited pitfall traps, sifting forest litter and processing it through Berlese funnels.

#### Oxypoda manitobae

Taxon classificationAnimaliaColeopteraStaphylinidae

Casey

(for illustrations, see Klimaszewski et al. 2006)

##### Distribution.

**Table:** 

Origin	Nearctic
Distribution	Canada: BC, MB, **SK**. USA: CO
New provincial records	CANADA, **Saskatchewan**, Larson Ranch, Hwy 21, 16 km S Maple Creek: 17-VI-2005, flood debris, D. Larson (DLC) 1 male; 15-30-VIII-2005, D. Larson (DLC) 1 female.
References	Casey 1911, Klimaszewski et al. 2006, Bousquet et al. 2013

##### Natural history.

In SK, specimens were captured in June and August, one male was found in flood debris along the margin of a seasonal creek. Elsewhere, adults were captured in July and August in Arctic habitats or in the Rocky Mountains (853-2896 m) (Klimaszewki et al. 2006).

#### Parocyusa fuliginosa

Taxon classificationAnimaliaColeopteraStaphylinidae

(Casey)

(for illustrations, see Klimaszewski et al. 2011, Brunke et al. 2012)

##### Distribution.

**Table:** 

Origin	Nearctic
Distribution	Canada: LB, ON, **SK**. USA: MA, NC, PA
New provincial records	CANADA, **Saskatchewan**, Larson Ranch, Hwy 21, 16 km S Maple Creek: 30-VIII-2014, D. Larson (DLC) 1 female.
References	As *Tetralecopora*: Casey 1906, Moore and Legner 1975, Seevers 1978; as *Parocyusa*: Klimaszewski et al. 2011, Brunke et al. 2012, Bousquet et al. 2013

##### Natural history.

In SK, one female was captured in August from unspecified habitat. In NF, adults were collected from rocks/gravel at a stream margin in early August (Klimaszewski et al. 2011).

### PLACUSINI Mulsant & Rey

#### Placusa incompleta

Taxon classificationAnimaliaColeopteraStaphylinidae

Sjöberg

(for diagnosis and illustrations, see Klimaszewski et al. 2001, 2011)

##### Distribution.

**Table:** 

Origin	Palaearctic, adventive in North America; possibly introduced separately in eastern Canada and western WA
Distribution	Canada: AB, BC, NB, NF, NS, ON, QC, **SK.** USA: WA; Palaearctic: Europe
New provincial records	CANADA, **Saskatchewan**, Cypress Hills Park, Center Block: Lodgepole Trail, 18-IX-2012, pine/spruce litter near stream, D. Larson (DLC) 1 male; fire guard, 29-X-2013, under fresh-cut pine slabs, D. Larson (DLC) 1 male; Sucker Creek, 1-VI-2012, under bark of recently killed aspen, D. Larson (DLC) 1 female.
References	Klimaszewski et al. 2001, 2011, Bousquet et al. 2013, Klimaszewski et al. 2015a

##### Natural history.

In SK, specimens were captured in pine/spruce litter near stream, under fresh-cut pine slabs, and under bark of recently killed aspen. In AB, adults were collected from dead or dying white spruce in aggregated retention patches surrounded by different levels of dispersed retention, using emergence traps and window traps (Klimaszewski et al. 2015a). Elsewhere, adults were found in various deciduous and coniferous forests, using a pit-light trap and ethanol-baited Lindgren funnel traps (Klimaszewski et al. 2001, 2011). The adults in northwestern Alberta were collected from June to September (Klimaszewski et al. 2015a).

#### Placusa pseudosuecica

Taxon classificationAnimaliaColeopteraStaphylinidae

Klimaszewski

(for diagnosis and illustrations, see Klimaszewski et al. 2001)

##### Distribution.

**Table:** 

Origin	Nearctic
Distribution	Canada: AB, BC, QC, ON, **SK**
New provincial records	CANADA, **Saskatchewan**, Cypress Hills Park, Center Block: fire guard, 29-X-2013, under fresh-cut pine slabs, D. Larson (DLC) 1 male, 1 female.
References	Klimaszewski et al. 2001, Bousquet et al. 2013, Klimaszewski et al. 2015a

##### Natural history.

In SK, adults were captured under fresh-cut pine slabs. In AB, adults were collected from dead or dying white spruce in aggregated retention patches surrounded by different levels of dispersed retention, using window traps (Klimaszewski et al. 2015a). Elsewhere, adults were found in mature coniferous forests, using pit-light traps and ethanol-baited Lindgren funnel traps (Klimaszewski et al. 2001). The adults were collected from July to August.

#### Placusa tachyporoides

Taxon classificationAnimaliaColeopteraStaphylinidae

(Waltl)

(for diagnosis and illustrations, see Klimaszewski et al. 2001)

##### Distribution.

**Table:** 

Origin	Palaearctic, adventive in North America
Distribution	Canada: AB, BC, NB, NS, QC, ON, **SK.** USA: CA, MA. Palaearctic: Europe, the Mediterranean, Caucasus, Siberia, Japan
New provincial records	CANADA, **Saskatchewan**, Larson Ranch, Hwy 21, 16 km S Maple Creek, 30-V-2014, D. Larson (DLC) 1 male; Cypress Hills Park, Center Block, Sucker Creek, 1-4-VI-2012, under bark of recently killed aspen, D. Larson (DLC) 1 male.
References	Moore and Legner 1975, Klimaszewski et al. 2001, Bousquet et al. 2013, Klimaszewski et al. 2015a

##### Natural history.

In SK, one male was captured under bark of recently killed aspen. In AB, adults were reared from white spruce logs in early and intermediate decay stages in white spruce dominated stands (Klimaszewski et al. 2015a). Elsewhere, adults were found in various deciduous and coniferous forests, using a flight intercept trap, ethanol-baited Lindgren funnel traps, pit-light traps, and pitfall traps (Klimaszewski et al. 2001).

#### Placusa tacomae

Taxon classificationAnimaliaColeopteraStaphylinidae

Casey

(for diagnosis and illustrations, see Klimaszewski et al. 2001)

##### Distribution.

**Table:** 

Origin	Nearctic
Distribution	Canada: AB, BC, NB, NF, NS, NT, QC, ON, **SK**, YT. USA: AZ, MA, WA, WI
New provincial records	CANADA, **Saskatchewan**, Larson Ranch, Hwy 21, 16 km S Maple Creek, 12-IX-2013, mouldy aspen log, D. Larson (DLC) 1 female; Cypress Hills Park, Center Block, fire guard: 10-IX-2013, newly cut lodgepole pine log, D. Larson (DLC, LFC) 3 males, 3 females; 8-VIII-2013, *Ips* tunnels in lodgepole pine (DLC) 3 males, 1 female; 26-VIII-2014, under bark of lodgepole pine (DLC) 1 male.
References	Casey 1893, Hatch 1957, Moore and Legner 1975, Klimaszewski et al. 2001, Webster et al. 2009, Klimaszewski et al. 2011, Bousquet et al. 2013

##### Natural history.

In SK, adults were captured from mouldy aspen log, newly cut lodgepole pine log, and in *Ips* tunnels in lodgepole pine. In eastern Canada, *Placusa tacomae* was collected in Lindgren funnel traps from *Pinus strobus*, *Pinus resinosa*, *Pinus banksiana*, *Picea glauca*, and *Acer saccharum* stands (Klimaszewski et al. 2001). In western Canada, a single individual of this species was recovered from an alpha-pinene-baited Lindgren trap at 850 m elevation in the coastal montane forest near Campbell River on Vancouver Island (Klimaszewski et al. 2001). One specimen from Colorado was taken at an elevation of 9600 ft (1 ft = 0.3048 m) from *Picea engelmannii* forest (Klimaszewski et al. 2001). Western host tree forest: *Pinus monticola*, mature *Tsuga heterophylla* – *Abies amabilis*, *Pinus contorta* (Klimaszewski et al. 2001). Collection period: May-August and October in British Columbia. Scolytid host: *Dendroctonus ponderosae* (Alberta); *Ips pini* (British Columbia) (Klimaszewski et al. 2001).

#### Placusa vaga

Taxon classificationAnimaliaColeopteraStaphylinidae

Casey

(for diagnosis and illustrations, see Klimaszewski et al. 2001)

##### Distribution.

**Table:** 

Origin	Nearctic
Distribution	Canada: BC, NB, NS, NT, QC, ON, **SK**, YT. USA: CA
New provincial records	CANADA, **Saskatchewan**, Cypress Hills Park, Lodgepole Trail, 18-IX-2012, under bark of lodgepole pine, D. Larson (DLC) 1 male.
References	Casey 1911, Moore and Legner 1975, Klimaszewski et al. 2001, Bousquet et al. 2013

##### Natural history.

In SK, one specimen was captured under bark of lodgepole pine. In QC, specimens were captured in *Abies balsamea* stands: old-growth stands, undetermined age stands, in *Picea glauca* stand, and *Populus tremuloides* with *Picea glauca* stand (Klimaszewski et al. 2001). All Quebec specimens except one (Multi-Pher 7 pitfall trap) were captured in Lindgren funnel traps baited with alpha-pinene and 95% ethanol, and with 70% ethanol as preservative (Klimaszewski et al. 2001). Collecting period: June to August.

### Tribe SILUSINI Fenyes

#### Silusa californica

Taxon classificationAnimaliaColeopteraStaphylinidae

Bernhauer

(for diagnosis and illustrations, see Klimaszewski et al. 2003, 2011)

##### Distribution.

**Table:** 

Origin	Nearctic
Distribution	Canada: AB, BC, NB, NF, NS, NT, QC, ON, **SK**, YT. USA: AK, CA, MN
New provincial records	CANADA, **Saskatchewan**, Cypress Hills Park, Center Block, fire guard: 8-VIII-2013, gilled mushrooms, D. Larson (DLC) 2 males, 1 female, 1 sex undetermined; 10-IX-2013, decaying mushrooms, D. Larson (DLC) 1 female; 10-VIII-2004, lodgepole pine, D. Larson (DLC) 1 sex undetermined; 18-VIII-2014, old polypore fungus on dead lodgepole pine stump, D. Larson (DLC) 2 males, 2 females, 3 sex undetermined; Cypress Hills Park, Lodgepole Trail, 21-VIII-2013, dry and decaying mushrooms, D. Larson (DLC) 2 males.
References	Bernhauer 1905, Klimaszewski and Winchester 2002, Klimaszewski et al. 2003, 2005, Majka and Klimaszewski 2010, Bousquet et al. 2013

##### Natural history.

In SK, adults were captured from gilled mushrooms, dry and decaying mushrooms, old polypore fungus on dead lodgepole pine stump and on lodgepole pine. Elsewhere, adults of *Silusa californica* were collected from July through September by means of passive pitfall traps, Luminoc pit-light traps, Malaise traps and by sifting forest litter, wet moss on forest floor, marten dung on moss, and mushrooms (Klimaszewski et al. 2003). Most specimens were captured in the passive pitfall traps. Adults occurred in coniferous (red spruce, Sitka spruce), mixed-wood (yellow birch/balsam fir), and unspecified deciduous forests (Klimaszewski et al. 2003). The Alberta specimens were collected in boreal mixed-wood forest, predominantly trembling aspen with a small amount of eastern balsam poplar, white birch, white spruce, and willow species (Klimaszewski et al. 2003). Five of the specimens were taken from old stands at least 100 years of age, nine were from mature stands 65 to 75 years of age, and three were from a recently harvested stand, 3 years of age (Klimaszewski et al. 2003). The specimens from the Carmanah Valley, Vancouver Island, British Columbia, were mainly captured in the forest interior, followed by fewer in the transition zone, and only two specimens were found in the clear-cut zone (Klimaszewski and Winchester 2002).

### Tribe TACHYUSINI Thomson

#### Brachyusa

Taxon classificationAnimaliaColeopteraStaphylinidae

Mulsant & Rey

##### Key to Canadian species of *Brachyusa*

**Table:** 

1	Median lobe of aedeagus with narrowly triangular apical part forming dorsally distinct angular projection in apical half of tubus in lateral view (see Fig. 5N, in Seevers 1978)	***Brachyusa americana* (Fenyes)**
–	Median lobe of aedeagus with narrowly triangular apical part without angular dorsal projection in apical half of tubus in lateral view (Figs 93, 100)	**2**
2	Body broad (Fig. 99); pronotal base strongly sinuate laterally (Fig. 99); median lobe of aedeagus with tubus extremely elongate (Fig. 100); male tergite VIII emarginate apically (Fig. 101); spermatheca L-shaped (Fig. 105)	***Brachyusa saskatchewanae* Klimaszewski & Larson, sp. n.**
–	Body moderately narrow (Fig. 92); pronotal base evenly arcuate (Fig. 92); median lobe of aedeagus with tubus moderately elongate (Fig. 93); male tergite VIII truncate apically (Fig. 94); spermatheca S-shaped (Fig. 98)	***Brachyusa helenae* Casey**

**Figures 92–98.**
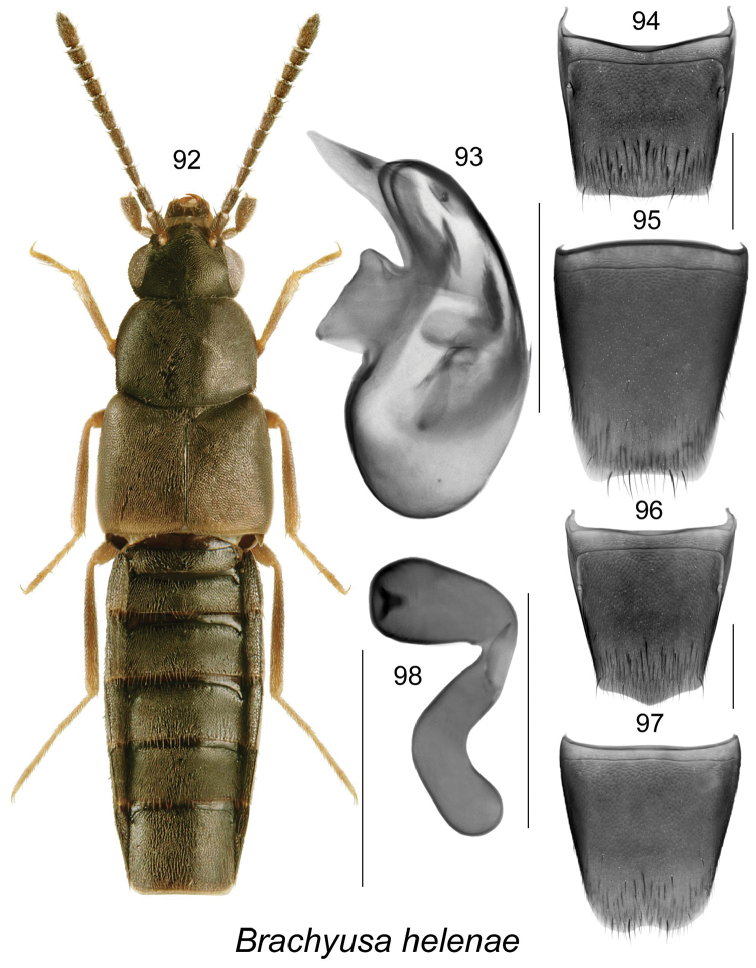
*Brachyusa helenae* Klimaszewski & Larson, sp. n.: **92** habitus in dorsal view **93** median lobe of aedeagus in lateral view **94** male tergite VIII **95** male sternite VIII **96** female tergite VIII **97** female sternite VIII **98** spermatheca. Scale bar for habitus = 1 mm, and the remaining scale bars = 0.2 mm.

**Figures 99–105.**
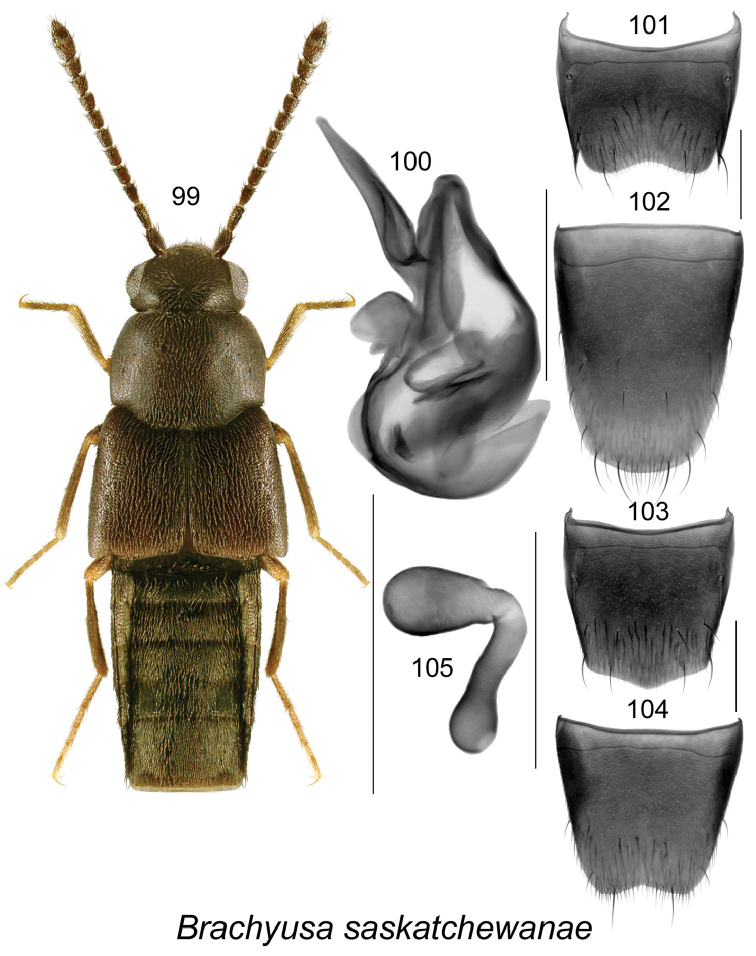
*Brachyusa saskatchewanae* Klimaszewski & Larson, sp. n.: **99** habitus in dorsal view **100** median lobe of aedeagus in lateral view **101** male tergite VIII **102** male sternite VIII **103** female tergite VIII **104** female sternite VIII **105** spermatheca. Scale bar for habitus = 1 mm, and the remaining scale bars = 0.2 mm.

#### Brachyusa helenae

Taxon classificationAnimaliaColeopteraStaphylinidae

(Casey)

Figs 92–98

(for diagnosis, see Klimaszewski et al. 2011)
Tetralina filitarsus Casey, 1911: 225. Holotype (male): USA, Montana, Kalispell, June, Wickham, Type USNM 3887 (USNM) 1 male. **New Synonymy**.

##### Distribution.

**Table:** 

Origin	Nearctic
Distribution	Canada: LB, NB, NF, NT, ON, **SK**, YT. USA: AK, MT
New provincial records	CANADA, **Saskatchewan**, Cypress Hills Park: Center Block, Lodgepole Trail, 18-IX-2012, pine/spruce litter near stream, D. Larson (LFC) 1 female; Loch Lomond, 29-VIII-2011, D. Larson (DLC) 1 female.
References	Casey 1911, Seevers 1978, Klimaszewski et al. 2011, Brunke et al. 2012, Bousquet et al. 2013

##### Natural history.

In SK, one specimen was captured in pine/spruce litter near stream, and another in an unspecified habitat in August and September. In LB, adults were collected in July and August on sand and gravel on the banks of the Churchill River (Klimaszewski et al. 2011). Elsewhere, adults were collected near lake and river shorelines, on clay, sand and gravel beaches and sandy and silty river margins (Klimaszewski et al. 2011). The adult activity period is May to August.

##### Comments.

The two SK females agree in colour, body shape, morphology of tergite and sternite VIII, and spermatheca with the type of *Brachyusa helenae* and the recently examined specimens from NF and NB. We have studied the types of *Brachyusa alutacea* (Casey), *Brachyusa filitarsis* (Casey) and *Brachyusa helenae* (Casey). The genital illustrations of *Brachyusa americana* (Fenyes), recorded from BC, are provided by Seevers (1978). We have not found any significant morphological differences between the types of *Brachyusa filitarsis* and *Brachyusa helenae*, and the two species are synonymous. However, *Brachyusa alutacea* clearly differs from *Brachyusa helenae*/*filitarsis* by a very broad body. Seevers’ (1978) key to species based on antennae and the length of the basal article of the metatarsus is not accurate.

#### Brachyusa saskatchewanae

Taxon classificationAnimaliaColeopteraStaphylinidae

Klimaszewski & Larson sp. n.

http://zoobank.org/B1B397E3-9706-4BD1-992E-B2210EE12B30

Figs 99–105

##### Holotype (male).

Canada, Saskatchewan, Bear Creek at Crane Lake, near Piapot, 18-VIII-2011, D. Larson (LFC). **Paratypes**. Canada, Saskatchewan, Grasslands National Park, Frenchman River at Ecotour Rd., 26-VII-2004, sandy-clay river bank, D. Larson (DLC) 1 male; Bigstick Lake, 16 km E Golden Prairie, 21-IX-2011, D. Larson (DLC, LFC) 4 females.

##### Etymology.

The name of this species, *saskatchewanae*-, is a feminine adjective derived from the name of the province of Saskatchewan, where the type series was found.

##### Diagnosis.

Body narrowly oval, length 2.3-2.5 mm, slightly flattened; uniformly black with light brown tarsi (Fig. 99); integument moderately glossy with short and silky pubescence (Fig. 99); antenna with articles I-VII elongate, VIII-IX subquadrate to slightly transverse (Fig. 99); head distinctly narrower than elytra and with large eyes, postocular region very short and abruptly narrowed basally (Fig. 99); pronotum wider than head but narrower than elytra, sinuate baso-laterally and strongly converging apically in apical third, pubescence directed straight and obliquely posteriad (Fig. 99); elytra at suture about as long as pronotum, pubescence directed straight posteriad, basal margin concave (Fig. 99); abdomen strongly narrowed posteriad, three basal tergites with deep transverse impressions (Fig. 99); metatarsus with basal article less than twice as long as second (Fig. 99). MALE. Tergite VIII transverse with broad apical emargination (Fig. 101); sternite VIII strongly elongate, with wide space between base of disc and antecostal suture, apical margin rounded (Fig. 102); median lobe of aedeagus with very long and narrow tubus in lateral view, bulbus large with large crista apicalis (Fig. 100). FEMALE. Tergite VIII slightly triangularly produced at apex (Fig. 103); sternite VIII with shallow apical emargination (Fig. 104); spermatheca L-shaped, with sac-shaped capsule angularly connected to club-shaped stem (Fig. 105).

*Brachyusa saskatchewanae* may be distinguished from other *Brachyusa* species by its uniformly black and narrow body, sinuate lateral margins of pronotum, and the genitalic features described above (Figs 99, 100, 105).

##### Distribution.

Known only from SK.

##### Natural history.

All SK specimens were captured near water with some on sandy-clay river bank. They were mainly collected by splashing water onto the bank, which caused the beetles to run up the bank.

#### Gnypeta minuta

Taxon classificationAnimaliaColeopteraStaphylinidae

Klimaszewski & Webster

(for diagnosis and illustrations, see Klimaszewski et al. 2008c)

##### Distribution.

**Table:** 

Origin	Nearctic
Distribution	Canada: NB, **SK**
New provincial records	CANADA, **Saskatchewan**, Cypress Hills Park, West Block, 5 km E AB border, 30-VI-2012, sandy-clay river bank, D. Larson (DLC) 1 female.
References	Klimaszewski et al. 2008c, Bousquet et al. 2013

##### Natural history.

In SK, one female was captured in June from sandy-clay river bank. In NB, two specimens were captured in June, one from under debris on muddy soil near a small pool in a silver maple forest, and the other from under debris on clay and sand mix at river margin (Klimaszewski et al. 2008c)

#### Gnypeta saccharina

Taxon classificationAnimaliaColeopteraStaphylinidae

Klimaszewski & Webster

(for diagnosis and illustrations, see Klimaszewski et al. 2008c)

##### Distribution.

**Table:** 

Origin	Nearctic
Distribution	Canada: NB, **SK**
New provincial records	CANADA, **Saskatchewan**, Grassland National Park, W Block, oxbow N jct Ecotour Tr-Frenchman River, 13-VI-2009, D. Larson (DLC) 2 males, 3 females; Grassland National Park, W Block, Ecotour stop 3, shallow oxbow pond, 11-VI-2009, D. Larson (DLC) 1 male, 2 females; Bigstick Lake, N Maple Creek, 4-VIII-2012, organic mud/sedges, rushes, etc. near water, D. Larson (DLC, LFC) 2 males, 4 females; Bigstick Lake, 16 km E Golden Prairie, 21-IX-2011, D. Larson (DLC, LFC) 4 males, 4 females; Larson Ranch, Hwy 21, 16 km S Maple Creek: 10-VI-1998, D. Larson (DLC) 1 male; dam, 28-VIII-2011, D. Larson (DLC) 1 male; 3-IX-2011, D. Larson (DLC) 1 male; Harris Res., 10 km S Maple Creek, wind-drift, 12-V-2012, D. Larson (DLC) 1 male; Cypress Hills Park, C Block, fire break, 10-VI-2011, under bark of lodgepole pine, D. Larson (DLC) 1 male; Cypress Lake, east dam, 12-VI-1998, D. Larson (DLC) 1 male; Cypress Lake Park, sifting wrack, 16-VI-2011, D. Larson (DLC) 1 male; Cypress Lake E end, sifting wrack, 31-VII-2012, D. Larson (DLC) 2 males, 1 female; Cypress Lake E dam, wind-drift, 9-V-2012, DE Larson (DLC) 1 male, 2 females.
References	Klimaszewski et al. 2008c, Bousquet et al. 2013

##### Natural history.

In SK, specimens were captured from May through September from shallow oxbow pond, organic mud/sedges, rushes, etc. near water, under bark of lodgepole pine, wind-drift, and by sifting wrack. In NB, adults were captured in May from moist leaves near margin of vernal pond in silver maple (*Acer saccharinum* L.) swamp, and in June from flood debris at the margin of the Saint John River (Klimaszewski et al. 2008c).

## Supplementary Material

XML Treatment for Aleochara (Echochara) elisabethae

XML Treatment for Aleochara (Xenochara) inexpectata

XML Treatment for Aleochara (Calochara) rubricalis

XML Treatment for Aleochara (Calochara) speculicollis

XML Treatment for Aleochara (Coprochara) suffusa

XML Treatment for Aleochara (Calochara) villosa

XML Treatment for Acrotona pseudopygmaea

XML Treatment for Acrotona subpygmaea

XML Treatment for Amischa analis

XML Treatment for Atheta (Dimetrota) crenuliventris

XML Treatment for Atheta (Dimetrota) districta

XML Treatment for Atheta (Dimetrota) pseudometlakatlana

XML Treatment for Atheta (Dimetrota) larsonae

XML Treatment for Atheta (Dimetrota) strigosula

XML Treatment for Atheta (Dimetrota) terranovae

XML Treatment for Atheta (Microdota) pseudopittionii

XML Treatment for Atheta (Microdota) riparia

XML Treatment for Atheta (Microdota) spermathecorum

XML Treatment for Atheta (Rhagocneme) subsinuata

XML Treatment for Atheta (Tetropla) frosti

XML Treatment for Atheta pseudoschistoglossa

XML Treatment for Atheta remulsa

XML Treatment for Atheta richardsoni

XML Treatment for Dinaraea angustula

XML Treatment for Dinaraea pacei

XML Treatment for Dochmonota

XML Treatment for Dochmonota langori

XML Treatment for Dochmonota simulans

XML Treatment for Dochmonota websteri

XML Treatment for Earota dentata

XML Treatment for Mocyta breviuscula

XML Treatment for Mocyta sphagnorum

XML Treatment for Nehemitropia lividipennis

XML Treatment for Philhygra falcifera

XML Treatment for Philhygra subpolaris

XML Treatment for Schistoglossa blatchlyei

XML Treatment for Strigota ambigua

XML Treatment for Strigota obscurata

XML Treatment for Autalia rivularis

XML Treatment for Falagria caesa

XML Treatment for Myrmecocephalus arizonicus

XML Treatment for Agaricochara pulchra

XML Treatment for Gyrophaena lobata

XML Treatment for Gyrophaena subnitens

XML Treatment for Leptusa gatineauensis

XML Treatment for Cypha crotchi

XML Treatment for Cypha inexpectata

XML Treatment for Oligota inflata

XML Treatment for Zyras obliquus

XML Treatment for Ganthusa eva

XML Treatment for Hylota ochracea

XML Treatment for Oxypoda demissa

XML Treatment for Oxypoda domestica

XML Treatment for Oxypoda irrasa

XML Treatment for Oxypoda manitobae

XML Treatment for Parocyusa fuliginosa

XML Treatment for Placusa incompleta

XML Treatment for Placusa pseudosuecica

XML Treatment for Placusa tachyporoides

XML Treatment for Placusa tacomae

XML Treatment for Placusa vaga

XML Treatment for Silusa californica

XML Treatment for Brachyusa

XML Treatment for Brachyusa helenae

XML Treatment for Brachyusa saskatchewanae

XML Treatment for Gnypeta minuta

XML Treatment for Gnypeta saccharina
